# Investigation on Reactivity and Selectivity of Electrocatalytic CO_2_ Reduction in Photochemically Synthesized Ag_19_ and Alloyed Ag_19_Cu_2_ and Ag_12_Cu_7_ Nanoclusters

**DOI:** 10.1021/acscentsci.5c00784

**Published:** 2025-07-07

**Authors:** Yu-Xin Wang, Jijie Li, Fu-Qiang Zhang, Zhikai Qi, Fengwei Zhang, Xian-Ming Zhang

**Affiliations:** † School of Chemistry and Material Science, 47842 Shanxi Normal University, Taiyuan 030031, Shanxi, China; ‡ Institute of Crystalline Materials, 12441 Shanxi University, Taiyuan 030006, Shanxi, China

## Abstract

Atomically precise nanoclusters are desirable for understanding the structure–property relationships in the electrocatalytic CO_2_ reduction reaction (eCO_2_RR), but suitable related models are lacking, especially those of low- or zerovalent noble metal nanoclusters and their alloyed analogues. We first developed a photochemical method toward silver nanocluster Ag_19_(4-^*t*^BuPhC≡C)_14_(Dpppe)_3_(SbF_6_)_3_ (**Ag**_**19**_-**2e**) and then related copper-doped alloyed nanocluster Ag_12_Cu_7_(4-^*t*^BuPhC≡C)_14_(Dpppe)_3_Cl_3_(SbF_6_)_2_ (**Ag**_**12**_ **Cu**_**7**_-**0e**). Herein, we present a larger alloyed nanocluster, Ag_19_Cu_2_(4-^*t*^BuPhC≡C)_16_(Dpppe)_4_(SbF_6_)_3_ (**Ag**_**19**_ **Cu**_**2**_-**2e**) and investigate the relationship between the structures and the eCO_2_RR performance of those related nanoclusters. The UV–vis and mass spectra revealed that **Ag**_**19**_ **Cu**_**2**_-**2e** forms via light-induced **Ag**_**19**_-**2e** generation followed by Cu­(II) attachment. eCO_2_RR tests showed that **Ag**_**19**_-**2e** is the least efficient, while its dicopper alloyed **Ag**_**19**_ **Cu**_**2**_ **-2e** favors formate, highlighting the important role of copper doping in regulating Ag cluster catalysis. This conclusion is further confirmed by the good catalytic performance of **Ag**_**12**_ **Cu**_**7**_-**0e**, which demonstrated the best C_1_ product selectivity for both CO and formate. Experimental and theoretical calculations indicate that its excellent catalytic performance is attributed to the removal of Cl ligands, exposing active Ag sites for launching the eCO_2_RR process. This work not only demonstrates that copper-doped silver nanoclusters significantly enhance catalytic activity but also reveals that varying copper doping levels enable modulation of product selectivity in eCO_2_RR.

## Graphical abstract

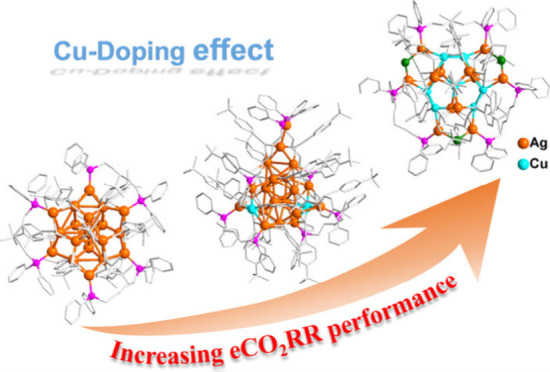

## Introduction

The electrocatalytic CO_2_ reduction reaction (eCO_2_RR) provides a promising approach to alleviate climate change by converting CO_2_ into value-added chemicals and fuels, thereby decreasing greenhouse gas emissions and facilitating the transition to a sustainable energy system.^1–9^ The eCO_2_RR involves multiple intricate steps, encompassing CO_2_ activation, proton-coupled electron transfer (PCET), formation and evolution of intermediates, and final product desorption. Catalyst selection critically guides the reaction through distinct pathways, significantly affecting product distributions. For instance, Cu-based catalysts exhibit a pronounced tendency toward multicarbon product formation, a behavior attributed to their uniquely adjustable electronic structure. Specifically, their tunable d-band center position enhances the stabilization of the critical *CO intermediate, thereby facilitating C–C coupling events. This stabilization capability underpins their catalytic selectivity, offering insights for advanced catalyst design targeting high-value chemical synthesis.^10–14^ However, understanding the eCO_2_RR mechanism at the atomic level is essential for designing efficient catalysts and optimizing the reaction activity. Among various categories of model catalysts, ligand-protected metal nanoclusters (NCs)^15–22^ have garnered significant attention due to their ultrasmall size, high specific surface area, and abundant active sites. Notably, their precise structures and compositions can be accurately determined through X-ray crystallography. These well-defined models provide unique advantages in elucidating the selectivity and reactivity of reactions, making them a rapidly growing area of research interest.^23–28^

On the other hand, alloying is an effective approach to tune the reactivity of a metal catalyst.^29–34^ Incorporating a second element can modify the electronic structure of surface active sites^35^ and optimize the adsorption strength of intermediates, promoting or inhibiting specific reaction pathways.^36^ Specifically, this electronic effect allows alloyed catalysts to more effectively stabilize key intermediates such as *COOH and *CO at the atomic level, lowering the activation barrier and improving conversion efficiency and selectivity.^37^ Therefore, alloyed NCs usually demonstrate superior catalytic performance compared to the corresponding homometallic counterparts.^38,39^ A significant amount of research has been conducted on Au-based NCs.^40–43^ For instance, monopalladium-doped Au_24_Pd NC showed substantially improved selectivity toward CO than Au_25_.^44^ A remarkable suppression of hydrogen evolution reaction (HER) is observed on Au_47_Cd_2_(TBBT)_31_ because of the doped Cd atoms.^45^ A similar effect is also found on Au_19_Cd_2_(SR)_16_.^46^ Fe atom tailored Au_8_ clusters enhanced the CO_2_ to CO activity by 18-fold as compared to Au_8_ NC.^47^ In this context, Ag–Cu alloyed nanoclusters have recently garnered attention due to their remarkable catalytic activity in eCO_2_RR, attributed to synergistic effects and plentiful active sites.^48–50^ For instance, the Ag_15_Cu_6_ cluster exhibits excellent catalytic activity for eCO_2_RR with FE_CO_ of 91.3%.^37^ In another study, one newly synthesized Ag_20_Cu_10_ cluster demonstrated significantly greater efficacy in facilitating eCO_2_RR compared to its counterparts.^51^ Despite these advances, systematic studies on Cu doped Ag NCs remain scarce, especially regarding doping-level effects, leaving a critical gap in understanding bimetallic synergies for the eCO_2_RR.

Herein, we bridge this gap by investigating a series of Ag–Cu nanoclusters with controlled Cu-doping levels. In the previous work, we reported a facile photochemical synthesis of two-electron reduced silver nanocluster Ag_19_(4-^*t*^BuPhC≡C)_14_(Dpppe)_3_(SbF_6_)_3_ (**Ag**_**19**_ **-2e**)^52^ and a Cu-doped alloyed nanocluster Ag_12_Cu_7_(4-^*t*^BuPhC≡C)_14_(Dpppe)_3_Cl_3_(SbF_6_)_2_ (**Ag**_**12**_ **Cu**_**7**_ **-0e**).^53^ Herein, a larger Ag–Cu bimetallic NC, Ag_19_Cu_2_(4-^*t*^BuPhC≡C)_16_(Dpppe)_4_(SbF_6_)_3_ (**Ag**_**19**_ **Cu**_**2**_ **-2e**), with two doped Cu atoms, are prepared. **Ag**_**19**_ **Cu**_**2**_ and previously reported **Ag**_**19**_ are both two-electron systems, providing ideal models to evaluate their eCO_2_RR performance. The result showed that **Ag**_**19**_ **-2e** is the least efficient while **Ag**_**19**_ **Cu**_**2**_ **-2e** is favorable for the formate, indicating that copper doping plays an important role in regulating the catalytic performance of Ag nanoclusters. The conclusion is further confirmed by the good catalytic performance of a higher degree of Cu doped **Ag**_**12**_ **Cu**_**7**_ **-0e** NC, which demonstrated the best C_1_ product for CO and formate in a wide potential range, where CO is the primary product. This work enriches the noble metal nanocluster catalyst models for eCO_2_RR and demonstrates the significance of Cu doping degrees on the reactivity and selectivity of eCO_2_RR at the atomic level.

## Results and Discussion

In a typical synthesis, AgSbF_6_ and Cu­(CF_3_COO)_2_ were dissolved in solvent before 4-*tert*-butylphenylacetylene (TBA) and 1,5-bis­(diphenylphosphino)­pentane (Dpppe) were introduced and then triethylamine (Et_3_N) was added. The reaction was initiated by irradiating the vessel with a 5 W white-light LED light.^52^ The color of the solution changed from an initial slight yellow to blue and eventually to red during the synthesis (Figure S1). After 24 h, the mixture was separated and subjected to a crystallization procedure to afford black block crystals in 5 days (Figures 1 and S2).

**Figure 1:**
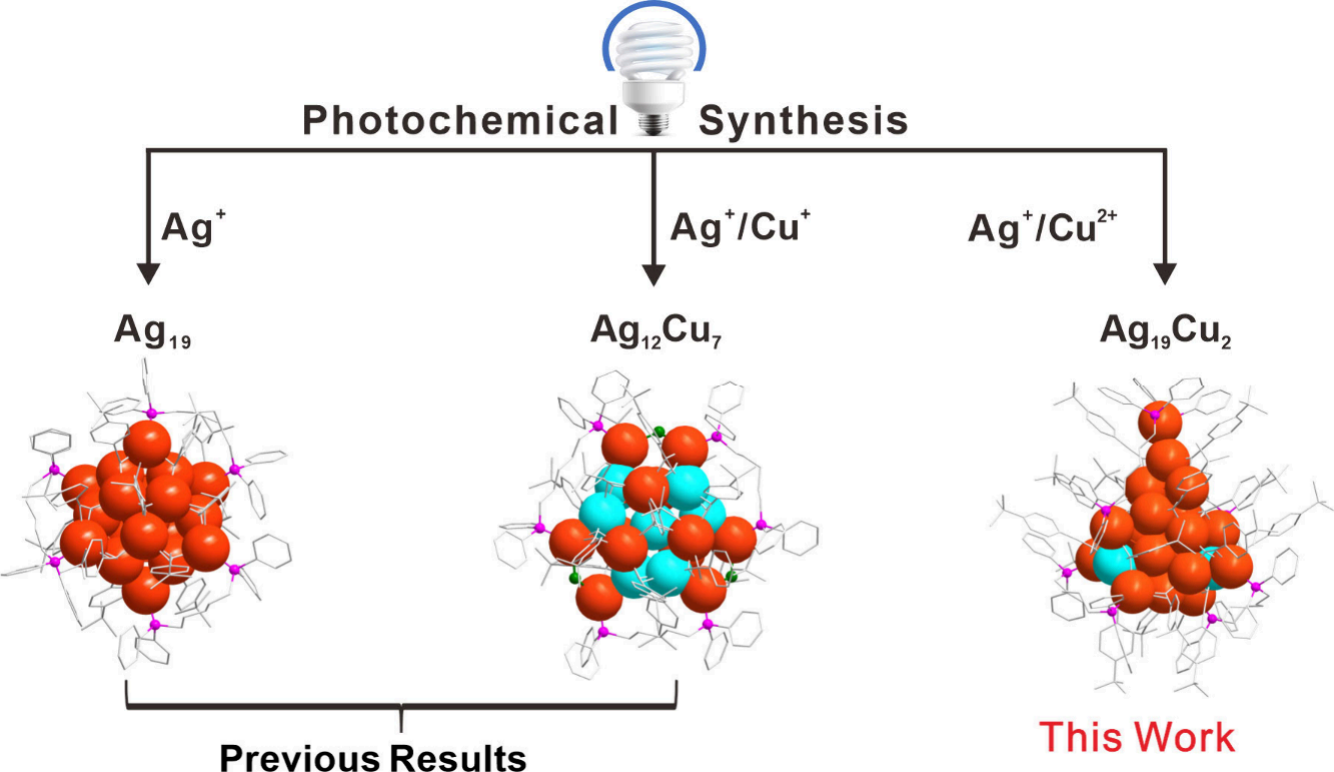
Previous results: photochemical synthesis of **Ag**_**19**_ and **Ag**_**12**_ **Cu**_**7**_ nanoclusters. This work: photochemical synthesis of atomically precise **Ag**_**19**_ **Cu**_**2**_ nanocluster.

Ag_19_Cu_2_(4-^*t*^BuPhC≡C)_16_(Dpppe)_4_(SbF_6_)_3_ NC (**Ag**_**19**_ **Cu**_**2**_) crystallizes in the *P*-1 space group (Figure S3). The thermal ellipsoid plot is shown in Figure S4. It consists of 19 Ag and two Cu atoms, which are protected by 16 alkyne and four Dpppe ligands (Figure 2a). Its structure can be described as a combination of a Ag_13_ core and two attached Ag_3_Cu motifs. The Ag_13_ core can be structurally divided into two distinct components: a triangular Ag_4_ unit and a fused structure comprising an Ag_6_ octahedron and an Ag_5_ triangular bipyramid, which share a common edge (Figure 2b). Two doped Cu atoms combine with the remaining six Ag atoms to form two Ag_3_Cu units, which are attached to both sides of the Ag_13_ core at waist positions. The Ag_13_ core and Ag_3_Cu are bridged by two Dpppe ligands, while the remaining two Dpppe ligands protect the Ag_3_Cu motifs. Ag_3_Cu motifs and Ag_13_ core are connected by alkyne ligands. Compared with **Ag**_**19**_ and **Ag**_**12**_ **Cu**_**7**_, **Ag**_**19**_ **Cu**_**2**_ exhibits richer alkynyl–metal coordination modes including μ_2_-η^1^, η^1^, μ_3_-η^1^, η^1^, η^1^, μ_3_-η^1^, η^1^, η^2^, μ_4_-η^1^, η^1^, η^1^, η^1^ and μ_4_-η^1^, η^1^, η^1^, η^2^ (Figure 2c).

**Figure 2:**
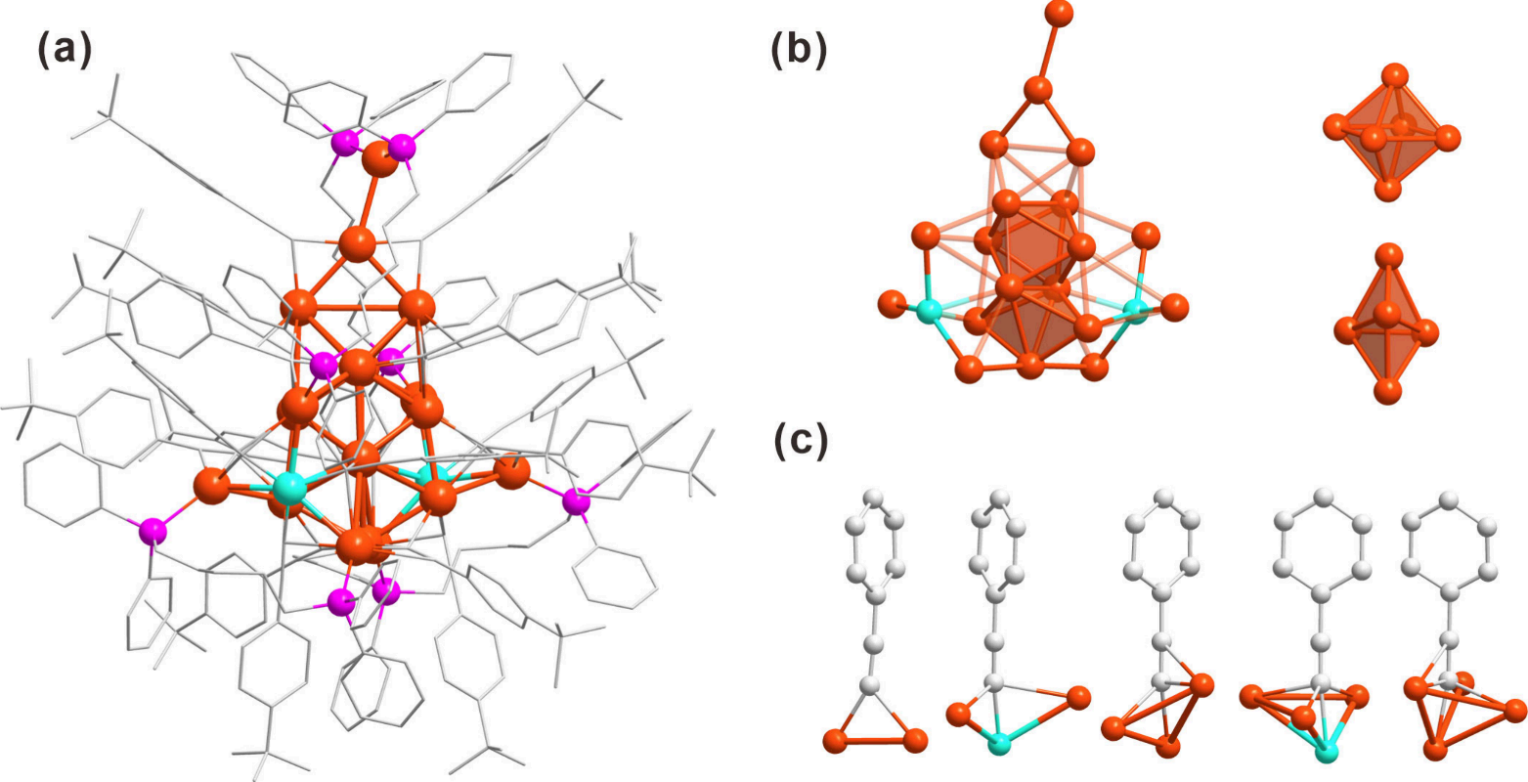
Crystal structure of Ag_19_Cu_2_(4-^*t*^BuPhC≡C)_16_(Dpppe)_4_(SbF_6_)_3_ (**Ag**_**19**_ **Cu**_**2**_). (a) A side view of **Ag**_**19**_ **Cu**_**2**_. (b) The core structure and the decomposition. (c) Coordination modes of alkyne ligands and metal atoms.

To confirm the molecular mass and composition of the cluster, electrospray ionization mass spectrometry (ESI-MS) was performed. As shown in Figure 3a, the ESI-MS spectrum of **Ag**_**19**_ **Cu**_**2**_ in positive mode exhibited a trication signal with the strongest intensity at 1798.70 Da, which corresponded to [Ag_19_Cu_2_(4-^*t*^BuPhC≡C)_12_(Dpppe)_2_(CF_3_CO_2_)_4_]^3+^ (calcd = 1798.72). The minor peak at 2815.52 Da was ascribed to [Ag_19_Cu_2_(4-^*t*^BuPhC≡C)_12_(Dpppe)_2_(CF_3_CO_2_)_4_(SbF_6_)_1_]^2+^ (calcd = 2815.54). The experimental isotopic patterns (red line) were in good agreement with the simulated ones (blue line). Additional peaks in the spectrum were attributed to the dissociation of **Ag**_**19**_ **Cu**_**2**_ in the solvent (Figures S5 and S6). According to the formula determined by ESI-MS and superatom theory, the **Ag**_**19**_ **Cu**_**2**_ cluster is a 2-electron closed-shell system (21_Ag/Cu_-16_ligands_-3_SbF^6–^_). The disappearance of the stretching mode of ν­(≡C–H) at 3300 cm^–1^ in the FT-IR spectrum of **Ag**_**19**_ **Cu**_**2**_ indicated the coordination of TBA with Ag (Figure 3b). The optical properties of **Ag**_**19**_ **Cu**_**2**_ were also characterized by ultraviolet–visible (UV–vis) spectra, showing two distinct peaks at 469 and 527 nm, respectively (Figure 3c).

**Figure 3:**
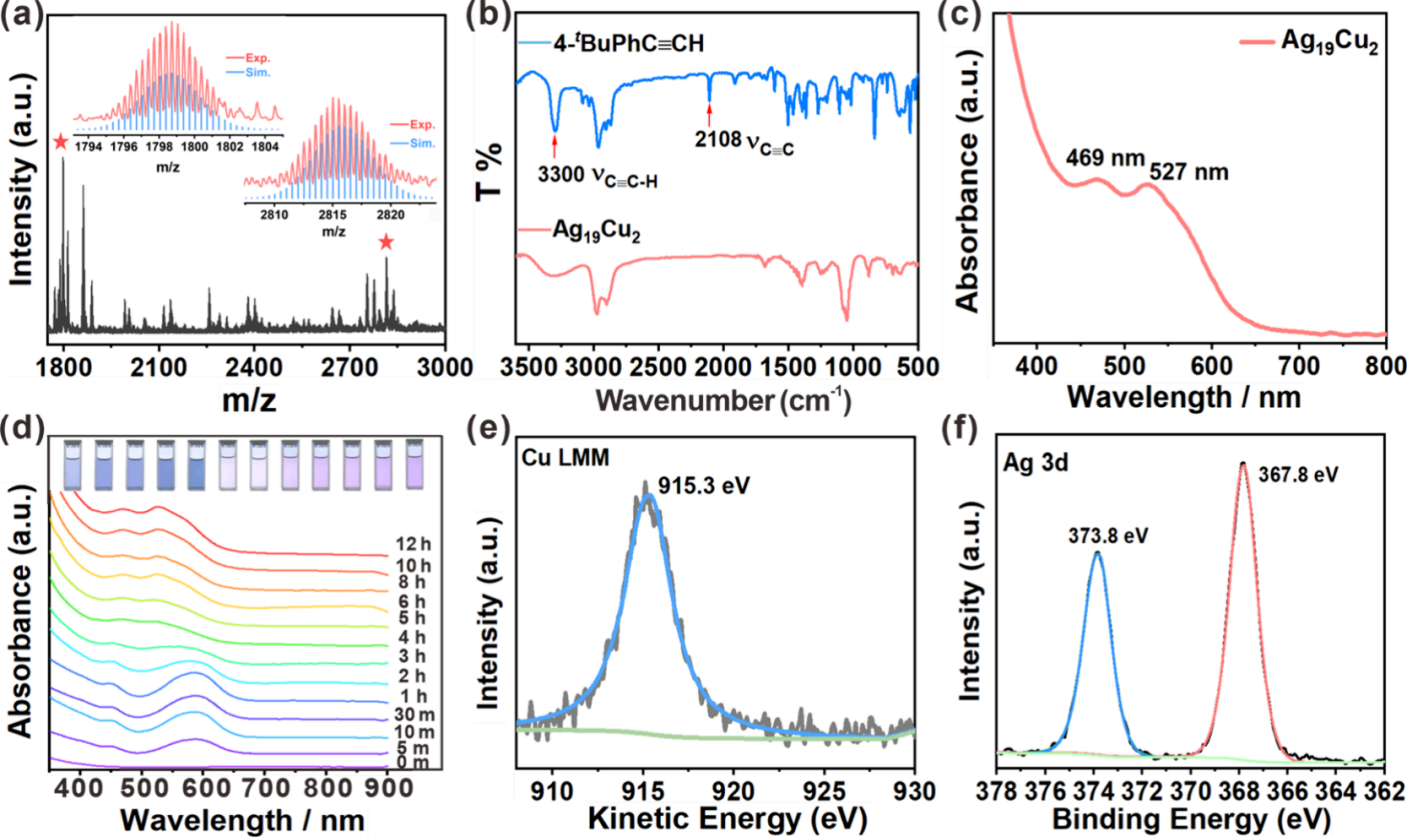
(a) Mass spectrum of Ag_19_Cu_2_(4-^*t*^BuPhC≡C)_16_(Dpppe)_4_(SbF_6_)_3_. Insets: experimental (red trace) and simulated (blue trace) isotopic patterns of the molecular ion peaks. (b) Infrared spectra of **Ag**_**19**_ **Cu**_**2**_ and TBA. (c) UV–vis absorption spectrum of **Ag**_**19**_ **Cu**_**2**_. (d) Time-course of UV–vis absorption during the synthesis of **Ag**_**19**_ **Cu**_**2**_. Reaction conditions: AgSbF_6_ (0.1 mmol), Cu­(CF_3_COO)_2_ (0.01 mmol), 4-^*t*^BuPhC≡CH (0.1 mmol), Dpppe (0.1 mmol) and Et_3_N (0.1 mmol). (e) XPS of the peak-fitted Cu LMM Auger spectrum and (f) the Ag 3d spectrum for **Ag**_**19**_ **Cu**_**2**_.

The synthesis process of **Ag**_**19**_ **Cu**_**2**_ was traced through the time course of UV–vis spectroscopy. As shown in Figure 3d, the initial state of the solution was featureless in the visible range. Upon irradiation, the solution developed a gradually increasing peak at 588 nm and a shoulder at 450 nm from 1 min to 1 h. These spectral features were characteristic of absorption of the **Ag**_**19**_ cluster NC (Figure S7). However, a notable divergence emerged from 2 h onward, and a blue shift to 469 and 527 nm was observed. The shifted peaks were characteristic of absorption of **Ag**_**19**_ **Cu**_**2**_. These results indicated that the synthesis of **Ag**_**19**_ **Cu**_**2**_ was a two-step process, in which light initially induced the formation of Ag NC that reacted with Cu­(II) species to yield **Ag**_**19**_ **Cu**_**2**_. In a control experiment, no evident change was discerned after a 12 h irradiation using only Cu­(CF_3_COO)_2_ as the precursor (Figure S8), proving **Ag**_**19**_ **Cu**_**2**_ originated from **Ag**_**19**_. In another experiment, pure **Ag**_**19**_ single crystals were dissolved in CH_2_Cl_2_, followed by the ethanol solution of Cu­(CF_3_COO)_2_ in a dropwise fashion. After 2 min, the color of the solution changed from purple to red, which was a characteristic color of **Ag**_**19**_ **Cu**_**2**_ (Figure S9). This also indicated that **Ag**_**19**_ **Cu**_**2**_ was indeed derived from the transformation of **Ag**_**19**_ by Cu incorporation. X-ray photoelectron spectroscopy (XPS) analysis was further conducted (Figures S10 and S11). As shown in Figure 3e, the Cu LMM Auger spectrum exhibits a prominent peak at 915.3 eV, corresponding to the Cu^+^ oxidation state. Additionally, the Ag 3d_5/2_ binding energy was 367.8 eV (Figure 3f), lower than that of bulk Ag (368.7 eV) and higher than that of Ag­(I) (367.3 eV). This confirmed that Ag atoms in **Ag**_**19**_ **Cu**_**2**_ exist in the Ag(0) and Ag­(I) oxidation states.

To gain deeper insight, the electronic structure of **Ag**_**19**_ **Cu**_**2**_ was examined by density functional theory (DFT) calculations. The frontier orbital charge density diagram and the partial density of states (PDOS) of the **Ag**_**19**_ **Cu**_**2**_ cluster were shown in Figure 4. Analysis of the total density of states (TDOS) in Figure 4a indicated that the top of the valence band was mainly composed of ligands, metal Ag 5s, and a few Cu 4s states. The bottom of the conduction band was dominated by Ag 5s and ligands. Figure 4b shows that the distribution of electron density of the highest occupied molecular orbitals (HOMOs) are primarily located on six Ag atoms in the core, suggesting that **Ag**_**19**_ **Cu**_**2**_ is a six-center two-electron (6c-2e) system, whereas the lowest unoccupied molecular orbitals (LUMOs) are mainly composed of some ligands and Ag_4_ units. Therefore, the charge transfer was assigned as a metal core to ligands charge transfer (MLCT) and metal to metal charge transfer (MMCT). Specifically, the HOMO–LUMO gap was 1.39 eV, which was close to the experimental value (Figure S12). The charge densities of other frontier orbitals are shown in Figure S13. Such electron-transfer characteristics provide possibility for photoluminescent behaviors. The photoluminescence of **Ag**_**19**_ **Cu**_**2**_ was then characterized. As shown in Figure S14, **Ag**_**19**_ **Cu**_**2**_ shows a prominent red emission in the range of 550–800 nm. The maximum excitation and emission wavelengths are 495 and 718 nm, respectively.

**Figure 4:**
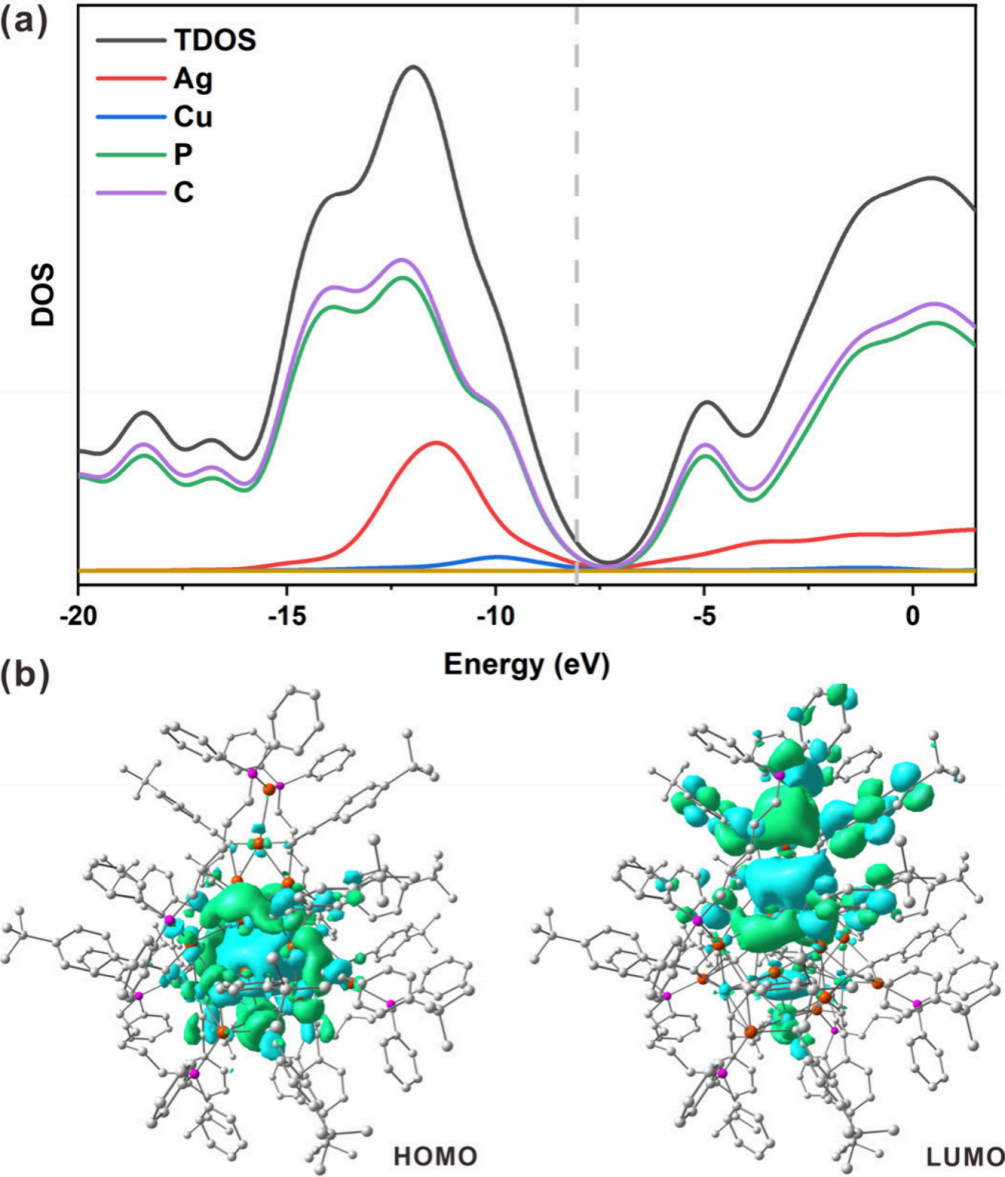
(a) Total and partial DOS of **Ag**_**19**_ **Cu**_**2**_ cluster. (b) Frontier molecular orbitals: HOMO and LUMO.

To investigate the impact of alloying on the electronic structures of clusters, the electronic energy levels of three NCs were compared. As shown in Figure 5a, the HOMO–LUMO energy gaps for **Ag**_**19**_, **Ag**_**19**_ **Cu**_**2**_, and **Ag**_**12**_ **Cu**_**7**_ clusters were calculated to be 1.16, 1.39, and 1.81 eV, respectively, indicating a significant increase of the gap upon the addition of Cu. Among them, the narrower HOMO–LUMO gap of **Ag**_**19**_ indicated easier electron transitions and demonstrated superior conductivity. Further, a quantitative analysis of molecular surfaces was conducted to compare the exposed site areas of three NCs for potential bonding interactions. The van der Waals surface area (WSA) analysis revealed changes in the accessible surface area of NCs, which directly affected the number of active sites available on the surface of the nanocluster in catalysis. Generally, a higher WSA led to enhanced catalytic activity because there were more sites for reactants to bind and undergo chemical transformations. The WSA analysis showed the calculated total WSA values were 3067.57 Å^2^ for **Ag**_**19**_, 3725.58 Å^2^ for **Ag**_**12**_ **Cu**_**7**_, and 3279.46 Å^2^ for **Ag**_**19**_ **Cu**_**2**_. Specifically, the WSA of 19 Ag sites in the **Ag**_**19**_ cluster was calculated to be 6.24 Å^2^, and 19 Ag and 2 Cu atoms in the **Ag**_**19**_ **Cu**_**2**_ cluster yielded a WSA value of 16.96 Å^2^, whereas 12 Ag and 7 Cu sites in the **Ag**_**12**_ **Cu**_**7**_ cluster were calculated to be 29.51 Å^2^. As a result, the WSA of Ag in **Ag**_**12**_ **Cu**_**7**_ is very high, indicating that Ag dominates on the cluster surface and provides a large number of active sites to promote the selective generation of CO. However, for the **Ag**_**19**_ **Cu**_**2**_ cluster, although the WSA of Ag is relatively high, it may not be sufficient to maintain an efficient CO generation pathway. Hirshfeld charge analysis on the **Ag**_**19**_ **Cu**_**2**_ cluster demonstrates that Cu atoms exhibit significantly higher positive charges compared to Ag atoms, which may promote the formation of formic acid (Figures S15 and S16). In **Ag**_**19**_, the Hirshfeld charge of the peripheral Ag atoms is higher than those in the Ag_13_ core, promoting the eCO_2_RR reaction (Figures S17 and S18). However, in the **Ag**_**12**_ **Cu**_**7**_ cluster, Ag atoms exhibit higher positive Hirshfeld charges than Cu atoms, except for those coordinated with Cl ligands, indicating that Ag atoms are likely the potential active sites (Figures S19 and S20).

**Figure 5:**
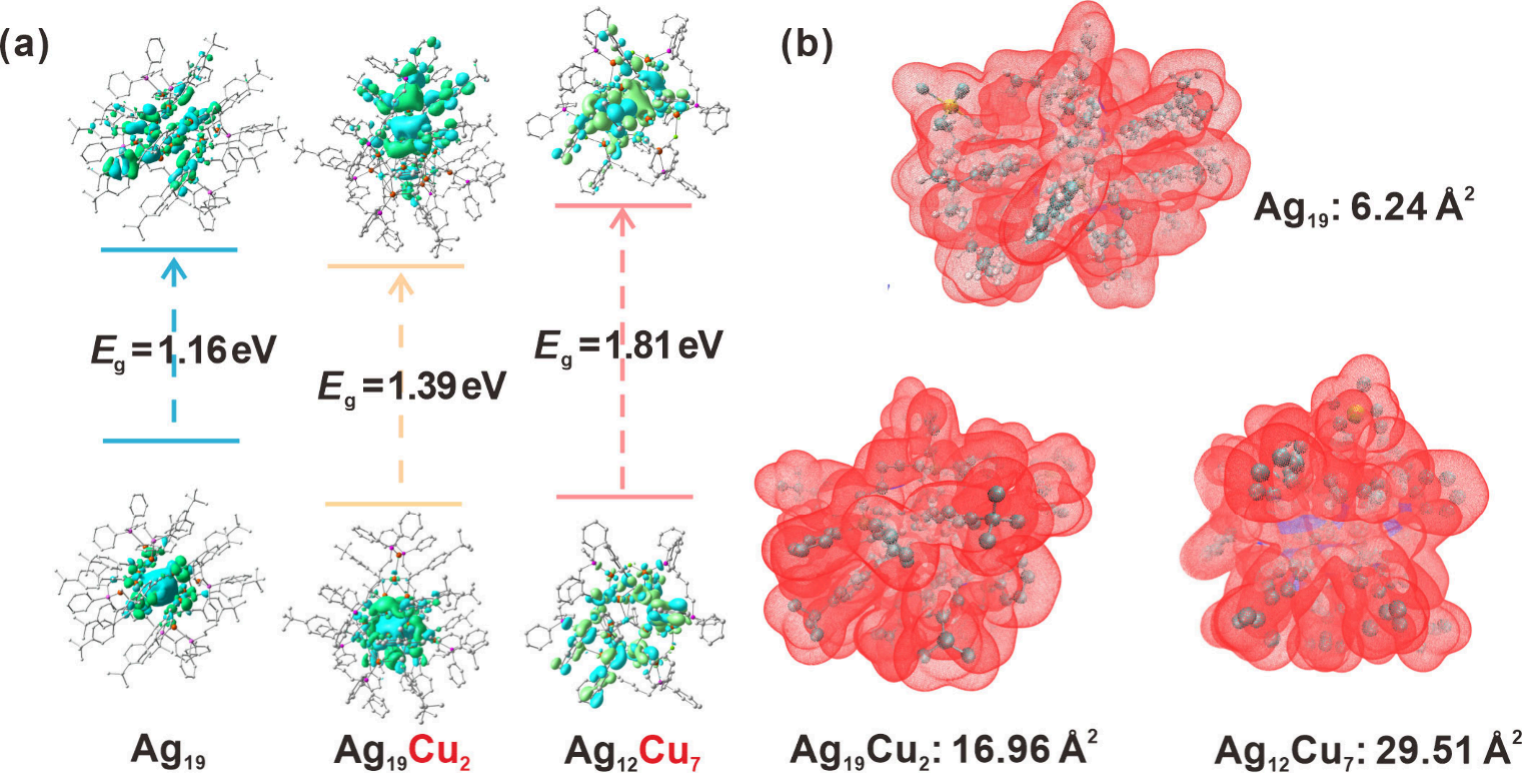
(a) Energy level diagrams and topology of HOMO and LUMO orbitals for **Ag**_**19**_, **Ag**_**19**_ **Cu**_**2**_, and **Ag**_**12**_ **Cu**_**7**_, respectively. (b) Visualization of the vdW surface areas of three nanoclusters_._.

To investigate the impact of Cu doping on the electrocatalytic performance in the eCO_2_RR, we initially focused on **Ag**_**19**_ and **Ag**_**19**_ **Cu**_**2**_ NCs. The activities of two NCs in eCO_2_RR were examined alongside **Ag**_**12**_ **Cu**_**7**_ to confirm that Cu doping can indeed modulate the eCO_2_RR performance by controlled potential electrolysis (CPE) measurements at various applied potentials in a flow cell (details in the Supporting Information). The gaseous and liquid products produced by eCO_2_RR were identified by gas chromatography (GC) and high-performance liquid chromatography (HPLC) as well as ^1^H NMR (Figure S21). The analysis demonstrated that only CO, formate, and H_2_ were identified, with no additional products detected. The linear scanning voltammetry (LSV) was conducted in 1.0 M KOH (pH = 14) solution under a high-purity CO_2_ environment. As shown in Figure 6a, **Ag**_**19**_ yielded the highest total current density among three NCs in the entire potential range due to the narrowest HOMO–LUMO gap. A current density of 10.2 mA cm^–2^ was achieved by **Ag**_**19**_, higher than that of **Ag**_**12**_ **Cu**_**7**_ (7.94 mA cm^–2^) and **Ag**_**19**_ **Cu**_**2**_ (6.53 mA cm^–2^) at −0.45 V vs reversible hydrogen electrode (RHE). The sum of Faradaic efficiency (FE) of CO and formate was 92.4% for **Ag**_**12**_ **Cu**_**7**_ at −1.17 V vs RHE, which displayed the best C_1_ product selectivity. Between them, **Ag**_**12**_ **Cu**_**7**_ showed remarkable eCO_2_RR selectivity for CO, delivering a FE_CO_ value of 71.2% at −1.17 V vs RHE (Figure 6b). In contrast, while the **Ag**_**19**_ cluster exhibited modest catalytic activity, the Cu-doped **Ag**_**19**_ **Cu**_**2**_ displayed significantly enhanced formate production, reaching a maximum FE_formate_ of 41.9% at −0.77 V vs RHE (Figure S22). This marked divergence underscores the critical role of Cu doping levels in steering the eCO_2_RR pathway toward distinct product selectivity. The partial current densities of CO and formate (*j*_CO_ and *j*_formate_) were calculated. As shown in Figure 6c, the *j*_CO_ of **Ag**_**12**_ **Cu**_**7**_ is superior to that of **Ag**_**19**_ and **Ag**_**19**_ **Cu**_**2**_ at applied potentials ranging from −0.57 to −1.27 V vs RHE, reaching 117.9 mA cm^–2^ at −1.17 V vs RHE. Additionally, the *j*_formate_ of **Ag**_**19**_ **Cu**_**2**_ was up to 19.6 mA cm^–2^, higher than both **Ag**_**19**_ and **Ag**_**12**_ **Cu**_**7**_ at −0.77 V vs RHE (Figure 6d). The CO production rate was also calculated based on the mass of the catalyst. At the optimum potential of −1.17 V vs RHE, **Ag**_**12**_ **Cu**_**7**_ produces CO from eCO_2_RR with a rate of 5.23 mol g^–1^ h^–1^, higher than that of **Ag**_**19**_ (3.41 mol g^–1^ h^–1^) and **Ag**_**19**_ **Cu**_**2**_ (2.76 mol g^–1^ h^–1^) (Figure S23). The Tafel plots more clearly revealed the reaction kinetics. **Ag**_**12**_ **Cu**_**7**_ has the lowest Tafel slope of 146 mV dec^–1^ at low overpotential compared to 150 mV dec^–1^ for **Ag**_**19**_ and 156 mV dec^–1^ for **Ag**_**19**_ **Cu**_**2**_ (Figure 6e). As a result, **Ag**_**12**_ **Cu**_**7**_ was more active and capable of converting CO_2_ into CO. In addition, the LSV curves of **Ag**_**12**_ **Cu**_**7**_ saturated in Ar and a CO_2_ atmosphere further prove its intrinsic catalytic activity toward the eCO_2_RR (Figure 6f). The long-term electrolysis experiment for 13,000 s using **Ag**_**12**_ **Cu**_**7**_ at −0.97 V vs RHE was further conducted. As shown in Figure S24, there was no obvious decay in both the current density and FE_CO_. The above results reveal that the Cu doping degree greatly affects the selectivity of the products. For **Ag**_**19**_ **Cu**_**2**_, the formate formation is significant; **Ag**_**12**_ **Cu**_**7**_ demonstrates higher CO Faraday efficiency over a wide range of potentials; and **Ag**_**19**_ is least efficient.

**Figure 6:**
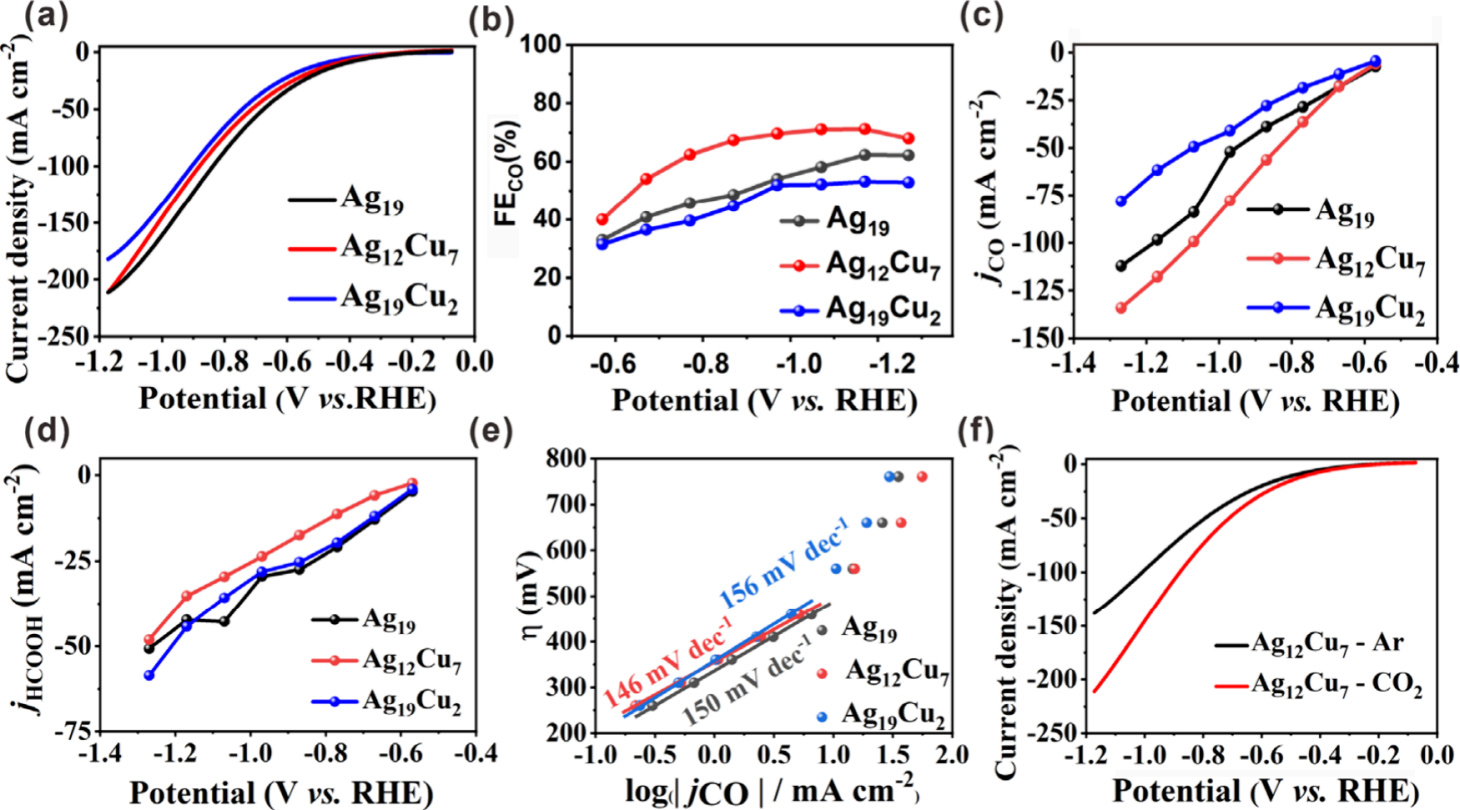
Electrochemical measurements for CO_2_ electroreduction in a flow cell. (a) Linear sweep voltammetry curves. (b) Faradaic efficiency of CO examined at different applied potentials. (c) CO partial current density in 1.0 M KOH. (d) HCOOH partial current density in 1.0 M KOH. (e) Tafel plots over **Ag**_**19**_, **Ag**_**12**_ **Cu**_**7**_, and **Ag**_**19**_ **Cu**_**2**_ NCs. (f) LSV curves of **Ag**_**12**_ **Cu**_**7**_ with Ar and CO_2_ at a scan rate of 10 mV s^–1^.

To further elucidate the differences of three clusters in eCO_2_RR activities, electrochemical impedance spectroscopy (EIS) and electrochemical active surface area (ECSA) measurements were performed, as illustrated in Figure 7. EIS results showed that the charge transfer resistance (*R*_ct_) of **Ag**_**12**_ **Cu**_**7**_ was lower than that of other catalysts during the eCO_2_RR, suggesting a more favorable electron transfer (Figure 7a). In addition, the ECSA of these catalysts was estimated using double-layer capacitance (*C*_dl_) (Figure S25). The *C*_dl_ of **Ag**_**12**_ **Cu**_**7**_ (0.052 mF·cm^–2^) was significantly higher than those of **Ag**_**19**_ (0.044 mF·cm^–2^) and **Ag**_**19**_ **Cu**_**2**_ (0.039 mF·cm^–2^), as evidenced by cyclic voltammetry measurements (Figure 7b). This result indicates that **Ag**_**12**_ **Cu**_**7**_ possesses the largest electrochemical active surface area (ECSA) among the tested materials, which could enhance the catalytic efficiency of the eCO_2_RR by providing more accessible active sites. To further understand the selectivity differences of three nanoclusters, we conducted XPS analysis. Among the three clusters, both **Ag**_**19**_ and **Ag**_**19**_ **Cu**_**2**_ are two-electron systems, and Ag has Ag(0) and Ag­(I) oxidation states. As shown in Figure S26, compared with **Ag**_**19**_, the +0.4 eV shift of Ag 3d in **Ag**_**19**_ **Cu**_**2**_ indicates electron transfer from Ag to Cu, creating Agδ^+^ and Cuδ^–^ sites and promoting the formation of formic acid. Since all Cu atoms in the **Ag**_**19**_ **Cu**_**2**_ and **Ag**_**12**_ **Cu**_**7**_ nanoclusters are in +1 oxidation states, we conducted a comparative analysis of their Cu elements using Auger electron spectroscopy. As shown in Figure S27, the Cu LMM Auger kinetic energy in **Ag**_**12**_ **Cu**_**7**_ exhibits a negative shift compared to that in **Ag**_**19**_ **Cu**_**2**_. This observation suggests a potential electron transfer from Cu to Ag in **Ag**_**12**_ **Cu**_**7**_, enhancing the production of CO. Herein, we have demonstrated that the selectivity of the product is determined not only by the type of doped metal but also by the doping level, providing important guidance for the further design and optimization of selectivity toward desired products in eCO_2_RR.

**Figure 7:**
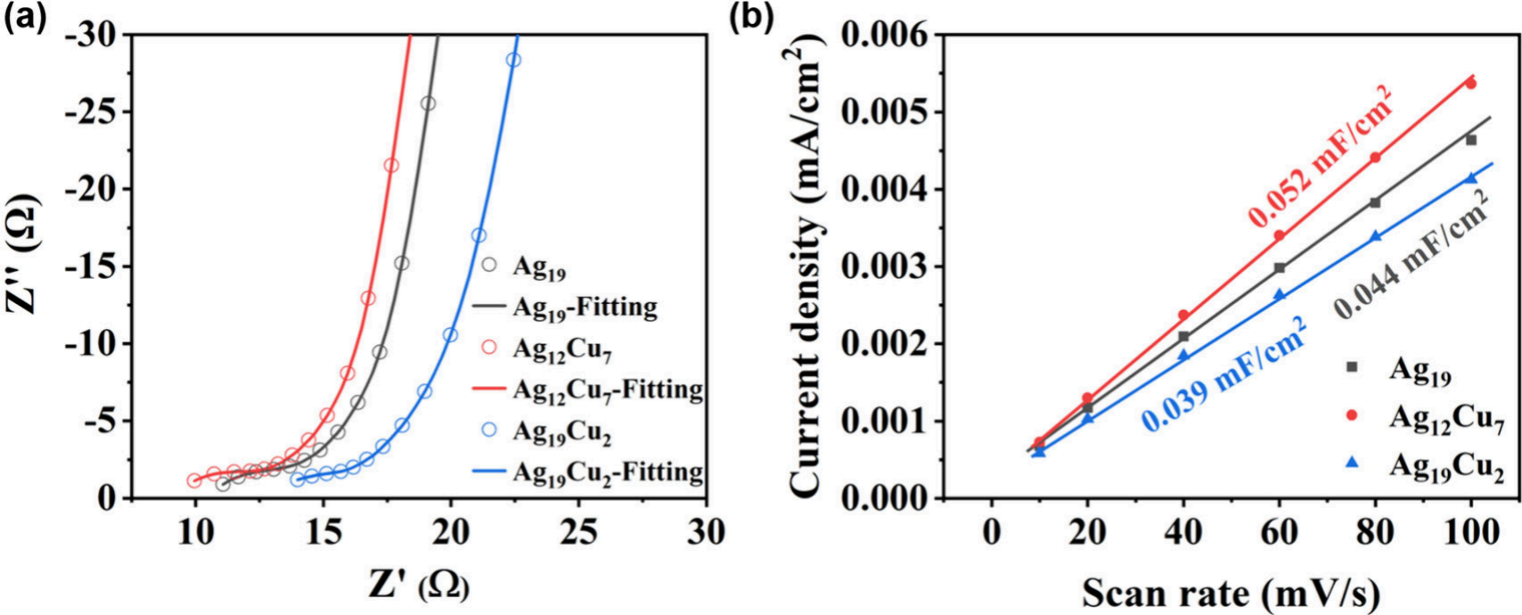
(a) Electrochemical resistance of **Ag**_**19**_, **Ag**_**12**_ **Cu**_**7**_, and **Ag**_**19**_ **Cu**_**2**_. (b) Slope analysis of electrochemically active surface area (ECSA)–electric double layer capacitance (*C*_dl_) correlation derived from cyclic voltammetry measurements.

We next performed DFT calculations to investigate the eCO_2_RR mechanism of the **Ag**_**12**_ **Cu**_**7**_ and **Ag**_**19**_ **Cu**_**2**_ clusters. It has been widely recognized that fully ligand-protected nanoclusters are typically inactive as eCO_2_RR catalysts.^54^ Therefore, the removal of ligands at a local site is considered to be crucial for the high electrocatalytic activity observed in nanoclusters. The ion chromatography analysis of the electrolyte in **Ag**_**12**_ **Cu**_**7**_ after the reaction detected the characteristic signal of chloride ion (Figure S28), confirming the removal of chlorides in the cluster-catalyst. Based on the experimental results, we calculated the pathway of the **Ag**_**12**_ **Cu**_**7**_ cluster with the removal of a Cl atom (Figure 8a). As shown in Figure 8b, the eCO_2_RR pathway analysis reveals that CO formation is governed by the *COOH → *CO conversion, which carries a reaction free energy of 0.26 eV. By contrast, HCOOH formation is limited by the *HCOO → *HCOOH step, with a significantly higher reaction free energy of 1.01 eV. Thus, **Ag**_**12**_ **Cu**_**7**_ favors CO production, in excellent agreement with the experiment. For the **Ag**_**19**_ **Cu**_**2**_ cluster, mass spectrometry analysis data of **Ag**_**19**_ **Cu**_**2**_ in Figure S5 indicated preferential alkyne ligand dissociation from Cu sites, exposing the Cu active sites. Accordingly, we simulated the eCO_2_RR pathway of the **Ag**_**19**_ **Cu**_**2**_ cluster with the removal of alkyne ligand on the Cu site (Figure 8c). Here, the *COOH → *CO step governing CO formation requires 0.72 eV, while HCOOH production via *HCOO → *HCOOH requires 0.64 eV (Figure 8d). This energy difference explains the experimentally observed selectivity toward formic acid.

**Figure 8:**
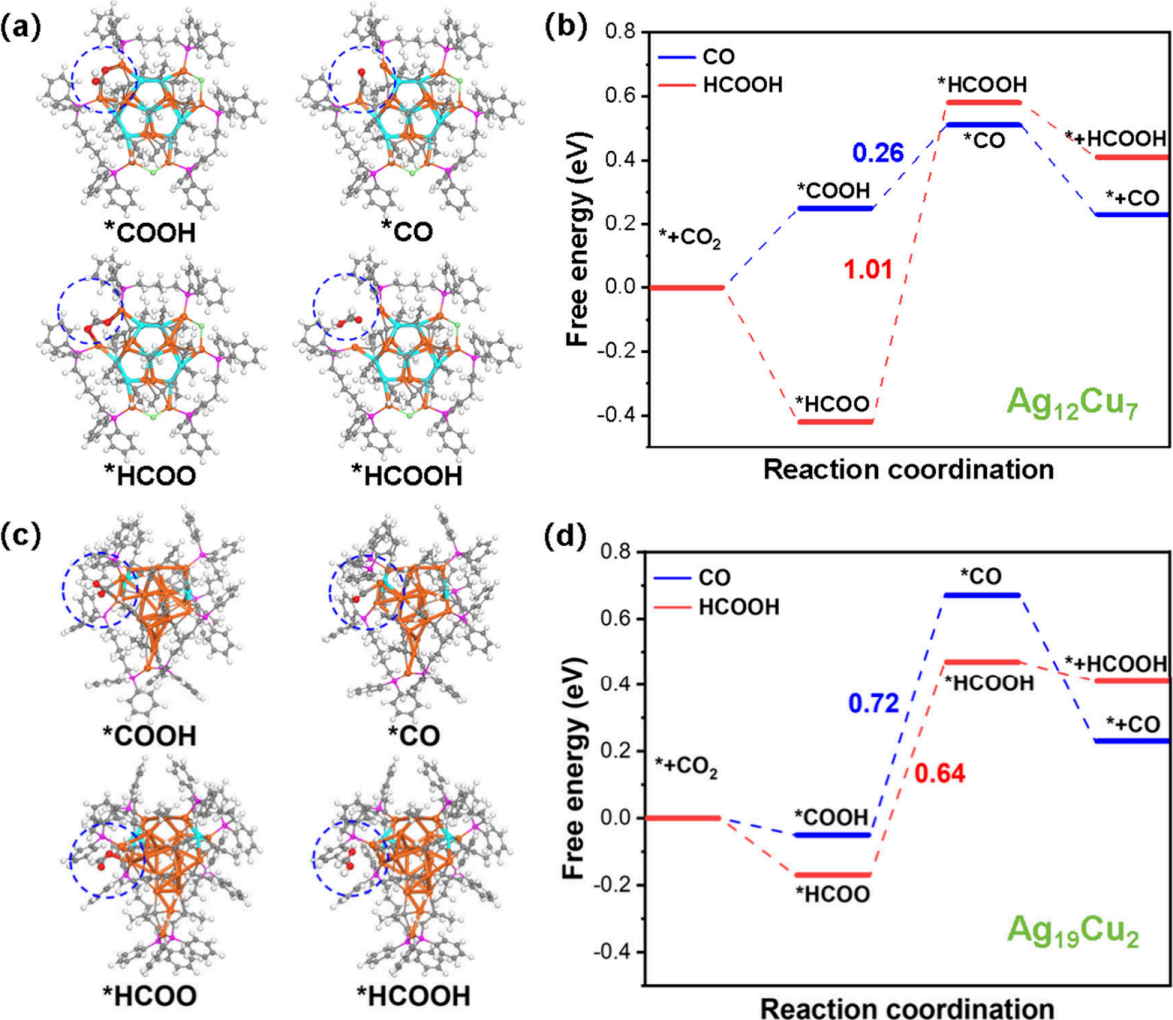
Optimized structures of key reaction intermediates involved in the eCO_2_RR of **Ag**_**12**_ **Cu**_**7**_ (a) and **Ag**_**19**_ **Cu**_**2**_ (c). Comparative free energy diagrams for CO and HCOOH production pathways on **Ag**_**12**_ **Cu**_**7**_ (b) and **Ag**_**19**_ **Cu**_**2**_ (d) at *U* = 0 V.

## Conclusions

In summary, the atomically precise alloyed nanocluster Ag_19_Cu_2_(4-^*t*^BuPhC≡C)_16_(Dpppe)_4_(SbF_6_)_3_ (**Ag**_**19**_ **Cu**_**2**_) is synthesized by a mild photochemical approach. The reaction mechanism and electronic structures are characterized by experiments and theoretical calculations. The catalytic performances of **Ag**_**19**_ **Cu**_**2**_, **Ag**_**12**_ **Cu**_**7**_ and **Ag**_**19**_ NCs in eCO_2_RR are evaluated in a flow cell. The results show that **Ag**_**12**_ **Cu**_**7**_ with a heavy Cu doping level has a higher selectivity for CO, **Ag**_**19**_ **Cu**_**2**_ has a decent performance and is more inclined for formate formation, whereas **Ag**_**19**_ is least efficient, suggesting that the Cu doping levels have an obvious regulation capability on the catalytic performance of single metal Ag nanoclusters. Theoretical calculations have provided valuable insights into the enhanced catalytic activity of the alloyed nanoclusters. We found that the Ag sites after stripping of Cl ligands from the intact **Ag**_**12**_ **Cu**_**7**_ cluster are critical for the formation of CO, while the high selectivity of **Ag**_**19**_ **Cu**_**2**_ for formic acid is attributed to the removal of alkyne ligands from Cu sites. This work advances photochemical methodology development while establishing a mechanistic framework to probe Cu-doping effects in Ag nanoclusters for the eCO_2_RR, offering critical insights for rationally engineering high-efficiency electrocatalysts.

## Methods

### Synthesis of Ag_19_Cu_2_ Nanocluster

AgSbF_6_ (0.1 mmol) and Cu­(CF_3_COO)_2_ (0.01 mmol) were dissolved in solvent before 4-^*t*^BuPhC≡CH (0.1 mmol) and 1,5-bis­(diphenylphosphino)­pentane (0.1 mmol) were introduced. Fourteen microliters of triethylamine (Et_3_N) was then added, followed by irridation with a 5 W white-light LED light. The color of the solution changed from the initial slight yellow to blue and eventually to dark red. After 24 h, the mixture was evaporated to dryness to give a dark red solid, which was dissolved in CH_2_Cl_2_ (2 mL) again. The solution was centrifuged for 10 min at 12,000 r/min. The suspension was precipitated with 10 mL of hexane, centrifuged at 12,000 r/min, and the supernatant was removed. The resulting red precipitate was dissolved in 2 mL of CH_2_Cl_2_ and then precipitated with 10 mL of hexane again. The CH_2_Cl_2_/hexane washing step was repeated four more times (*V*_DCM_:*V*_Hex_ = 1:5). Finally, the solution was subject to the diffusion of a mixture of ether and *n*-hexane (*V*:*V* = 1:1) at 4 °C to afford black block crystals in 5 days (isolated yield: 25%).

### Computational Details

The calculations were performed by using Gaussian16 software. The optimization of structures and HOMO/LUMO molecular orbitals were computationally studied using DFT. All calculations were carried out with the PBEPBE functional and the DEF2SVP basis set. The van der Waals (VDW) forces were calculated by utilizing Multiwfn software. Specifically, initially, a comprehensive quantitative analysis of three clusters was conducted, followed by a detailed examination of their electrostatic potential (ESP). Then, electron density with an iso value of 0.00100 was selected to define the real space function. Subsequently, the molecular vdW surface was defined by employing an isosurface value of 0.002 au. Finally, the corresponding regions of Ag and Cu nuclei on the molecular surface were visualized using Visual Molecular Dynamics (VMD) software with the locsurf.pdb file. In this context, the red region signifies the overall van der Waals surface area, while the blue region indicates the van der Waals surface area specific to the Ag or Cu metal atoms.

## Supplementary Material





## Notes

Additional structural and property characterizations, including materials and physical measurements, X-ray single-crystal analyses, control experiments, and DFT theoretical calculations (PDF)
Crystal structure of Ag_19_Cu_2_(4-^*t*^BuPhC≡C)_16_(Dpppe)_4_(SbF_6_)_3_ (CIF)

## Notes

Deposition Number 2395073 contains the supplementary crystallographic data for this paper. These data are provide free of charge by the joint Cambridge Crystallographic Data Centre and Fachinformationszentrum Karlsruhe Access Structures service www.ccdc.cam.ac.uk/structures.
