# Supplementary material for: Investigation on Reactivity and Selectivity of Electrocatalytic CO2 Reduction in Photochemically Synthesized Ag19 and Alloyed Ag19Cu2 and Ag12Cu7 Nanoclusters
Source: ACS Cent Sci. 2025 Jul 7;11(8):1428–37. doi: 10.1021/acscentsci.5c00784 (PMC12395295; doi:10.1021/acscentsci.5c00784)
Supplement: Supplementary file 1 [file oc5c00784_si_001.pdf]

Supporting Information

Investigation on Reactivity and Selectivity of Electrocatalytic CO_2 Reduction in Photochemically Synthesized Ag_{19} and Alloyed $Ag_{19}Cu_2$ & $Ag_{12}Cu_7$ Nanoclusters

Yu-Xin Wang[a,b‡], *Jijie Li*[b‡], *Fu-Qiang Zhang*[a], *Zhikai Qi*[a], *Fengwei Zhang*[b], *Xian-Ming Zhang*[a,b*]

[a]School of Chemistry and Material Science, Shanxi Normal University, Taiyuan, 030006, Shanxi

[b]Institute of Crystalline Materials, Shanxi University, Taiyuan, 030031, Shanxi

[‡]These authors contributed equally to this work.

*Corresponding Authors: zhangxm@dns.sxnu.edu.cn; zhangxianming@tyut.edu.cn.

Materials and reagents.

Silver hexafluoroantimonate ($AgSbF_6$, 98%), Copper(II) trifluoroacetate ($Cu(CF_3COO)_2$, 98%), 4-tert-Butylphenylacetylene (4-^{t}BuPhC≡CH, 98%) and 1,5-Bis(diphenylphosphino)pentane (Dpppe, 98%) were purchased from Saen Chemical Technology (Shanghai) Co., Ltd. Triethylamine (Et_3N, 99.5%) was purchased from Anhui Zesheng Technology Co., Ltd. All solvents were purchased from Tianjin Damao Chemical Reagent Factory. All chemicals and solvents were used as received.

METHODS

1. Physical measurements

FT-IR spectra were collected on Nicolet iS5 with samples prepared as KBr pellets. UV-vis absorption spectra were recorded on TU-1950 UV-vis spectrophotometer with samples dispersed in CH_2Cl_2.The X-ray photoelectron spectra (XPS) were measured on a Thermo Fischer model Thermo Scientific K-Alpha+ XPS system from a

monochromatic aluminum anode X-ray source and the C1s line at 284.8 eV is used as the binding energy reference.

2. X-ray single-crystal analyses

Single crystals were selected in an optical microscope and protected by vacuum grease. Cell parameters and intensity data were recorded on a Rigaku Oxford Diffraction XtaLABSynergy-Sdiffractometer equipped with a HyPix-6000HE Hybrid Photon Counting (HPC) detector and an Oxford Cryosystems CryostreamPlus 800 open-fow N2 cooling device at 150 K using CuKα (λ=1.54184 Å). Absorption corrections were applied by using the program CrysAlis (multi-scan). The structure was solved and refined using Full-matrix least-squares based on F^2 with program SHELXS and SHELXL within OLEX2. Non-hydrogen atoms were refined anisotropically. All remaining hydrogen atoms were added at calculated positions and refined using riding models with isotropic displacement parameters based on those of the parent atoms.[1-3] Appropriate restraints or constraints were applied to the geometry and the atomic displacement parameters of the atoms in the cluster. The structure was examined using the Addsym subroutine of PLATON to ensure that no additional symmetry could be applied to the models. Since the crystals have very weak diffraction, especially in higher angles, the value of sine(theta_max)/wavelength is less than 0.575. Although a high-brilliance luS microfocus with Cu radiation was used reasonable diffraction data could be obtained only to moderate resolution, despite multiple attempts to improve crystal quality. Pertinent crystallographic data collection and refinement parameters are collated in Supplementary Table 1.

3. Electrochemical CO_2 reduction measurements

The working electrode is a carbon paper electrode coated with the sample. The process is as follows. 5 mg nanocluster and 5 mg XC -72 carbon black were dispersed in ethanol (1 mL) solution with 40 μL of Nafion. Then, the mixture was ultrasonicated for 40 min to obtain a suspension. 450 μL of the suspension was coated on carbon paper.

All electrochemical tests were carried out on a CHI 660E potentiostat at ambient temperature and pressure in a three-electrode system. A platinum sheet was used as the

counter electrode, and saturated Ag/AgCl was applied as the reference electrode. The electrolyte was a 1 M $KHCO_3$ aqueous solution, and a flow rate of 20 sccm CO_2 was maintained through the cathodic electrolyte during the whole test. Linear sweep voltammetry (LSV) curves were recorded on a CHI 660E electrochemical workstation in CO_2-saturated 1 M KOH (pH = 14) at a scan rate of 10 mV s^{-1} from −1.1 to −2.2 V *vs.* Ag/AgCl. All potentials in this study were converted to the reversible hydrogen electrode (RHE) scale by the following equation:

$$E_{RHE} = E_{Ag/AgCl}\,(0.197\text{ V}) + E_{Ag/AgCl} + 0.0591 \times \text{pH}.$$

The FEs and partial current density were calculated as the formulas:

$$FE_{CO} = C_{CO} \times S \times (2FP/\text{RT}) / I_{total} \qquad FE_{H2} = C_{H2} \times S \times (2FP/RT) / I_{total}$$

$$FE_{formate} = C_{formate} \times V \times 2F / (I_{total} \times \text{t})$$

$$j_{CO} = C_{CO} \times S \times (2FP/RT) / A \qquad j_{H2} = C_{H2} \times S \times (2FP/RT) / A$$

$$j_{formate} = C_{formate} \times V \times 2F / (A \times \text{t})$$

where C_{CO}, and C_{H2} refer to the concentration of CO and H_2 measured by GC, C_{HCOOH} refer to the concentration of formate measured by HPLC, S (flow rate of CO_2) = 20 sccm, F (Faraday constant) = 96485 C mol^{-1}, P (atmospheric pressure) = 101300 Pa, R (gas constant) = 8.314 J·mol^{-1}·K^{-1}, T (thermodynamic temperature = 298.15 K, A refers to the geometric area of the electrode, I_{total} refers to the measured current, t refers to the electrolytic duration.

Electrochemical impedance spectroscopy (EIS) measurements were carried out by applying an AC voltage with 10 mV amplitude in a frequency range from 10^6 Hz to 0.1 Hz. The double-layer capacitance (C_{dl}) was determined by cyclic voltammetry (CV) to compare the electrochemical active area (ECSA) of different clusters.

4. eCO_2RR Computational details

Density functional theory (DFT) calculations were performed using the Vienna ab initio simulation package (VASP) under periodic boundary conditions.[4] The **$Ag_{12}Cu_7$** and **$Ag_{19}Cu_2$** clusters were centered in a cubic supercell (30 Å × 30 Å × 30 Å) and fully relaxed until forces fell below 0.02 eV/Å. Exchange-correlation interactions were

described by the Perdew-Burke-Ernzerhof (PBE) functional within the generalized gradient approximation (GGA).[5] The projector-augmented wave (PAW) method described core–valence interactions,[6] using a plane-wave cutoff energy of 350 eV. K-space sampling was performed at the Γ point, and van der Waals interactions included via the DFT-D3 correction scheme.[7]

Reaction free energy changes were evaluated using the computational hydrogen electrode (CHE) model proposed by Nørskov and co-workers, with the free energy change ΔG expressed as:

$$\Delta G = \Delta E + \Delta E_{ZPE} - T\Delta S + \Delta G_{pH} + \Delta G_{U}$$

where, ΔE, ΔE_{ZPE}, $T\Delta S$ represent the changes in electronic energy, zero-point energy, entropy at T = 298.15K, respectively, while ΔG_{pH} and ΔG_{U} (with ΔG_{U} = -eU) account for the free energy contributions due to proton concentration (pH = 0 under acidic conditions) and electrode potential (U).

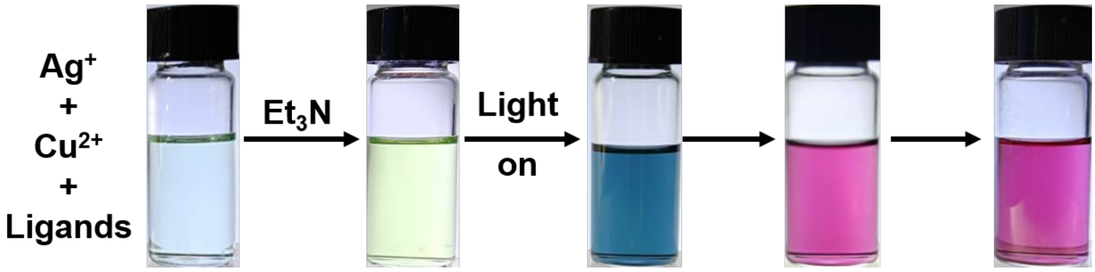

Figure S1. Photographs during photochemical synthesis of **$Ag_{19}Cu_2$**. Reaction conditions: $AgSbF_6$ (0.1mmol), $Cu(CF_3COO)_2$ (0.01mmol), 4-^{t}BuPhC ≡ CH (0.1 mmol), Dpppe (0.1 mmol) and Et_3N (0.1 mmol). Dpppe is 1,5-bis(diphenylphosphino)pentane. Irradiating the reaction solution by a 5-watt LED light for 24 hours.

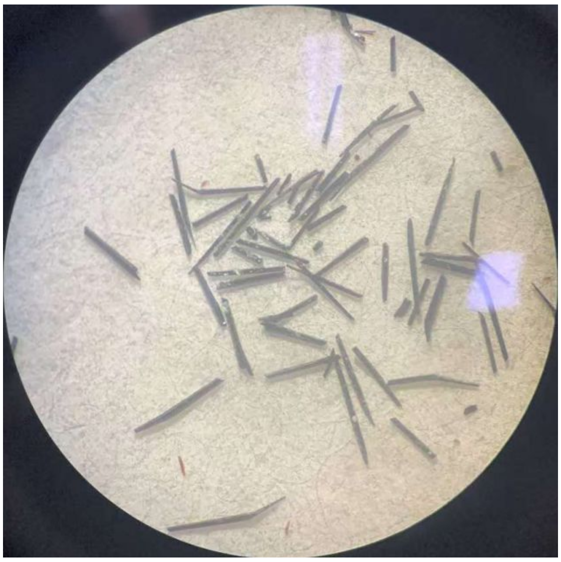

Figure S2. Photograph of **$Ag_{19}Cu_2$** single crystals.

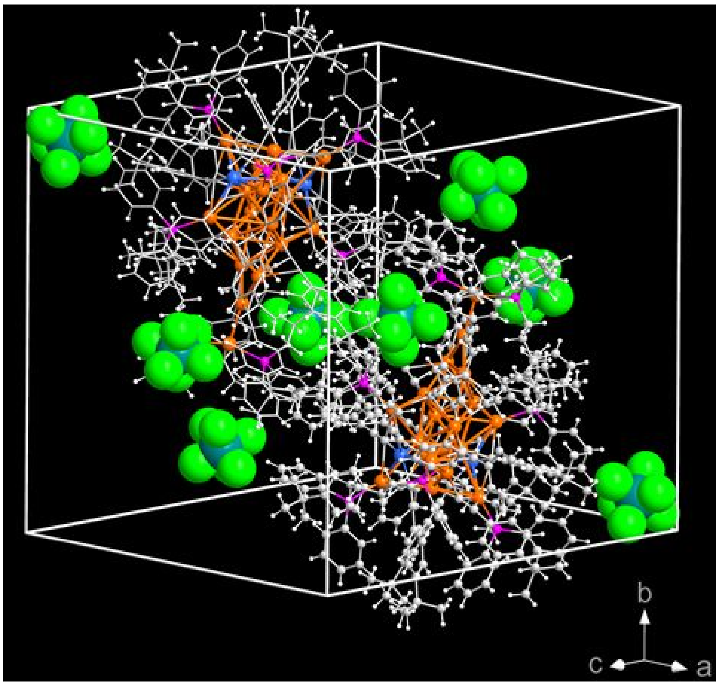

Figure S3. Packing of $\mathbf{Ag_{19}Cu_2}$ clusters in a unit cell. Color legend: Ag, orange; Cu, turquiose; P, green; F, bright green; Sb, teal; C, gray.

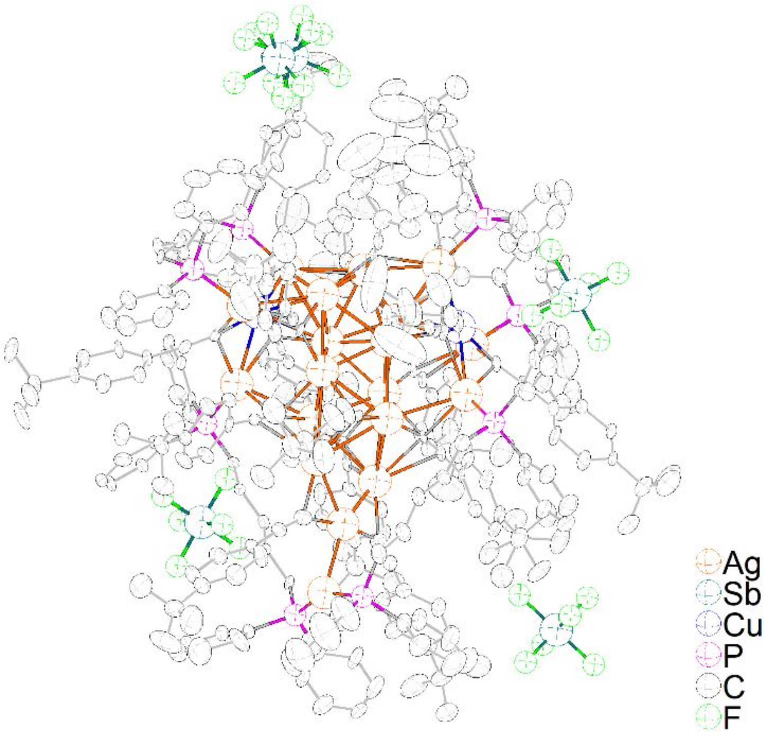

Figure S4. Thermal ellipsoid plot of $\mathbf{Ag_{19}Cu_2}$.

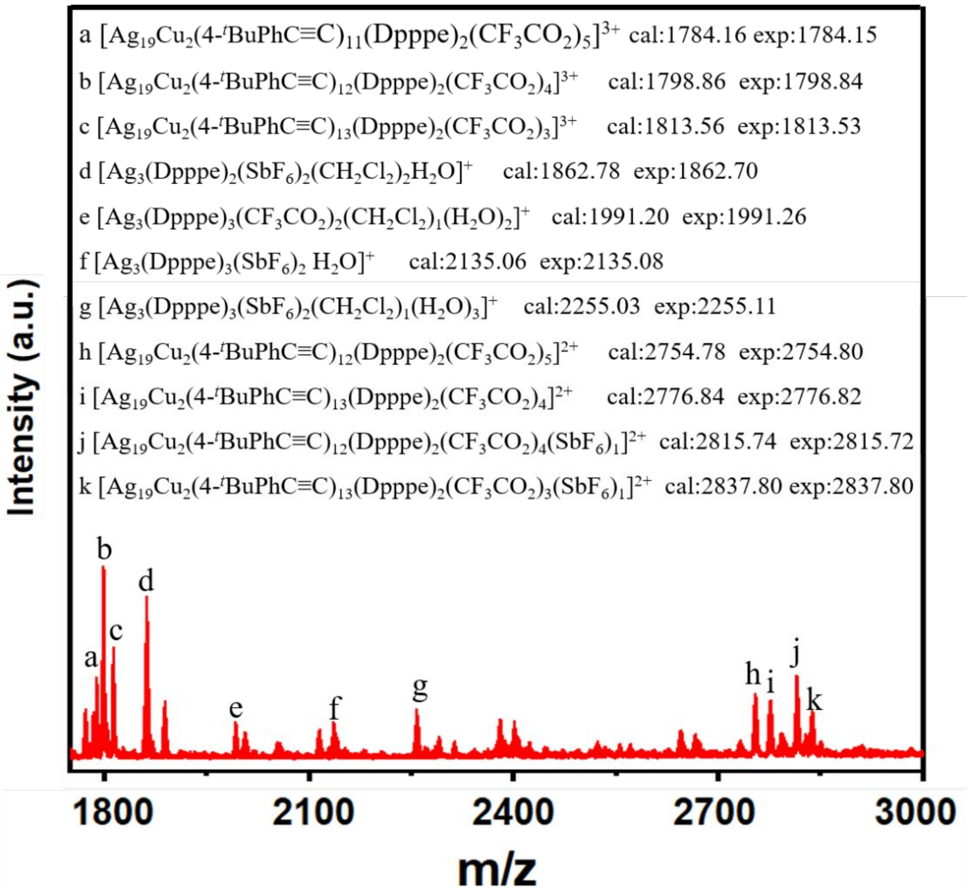

Figure S5. Mass spectrum of $\mathbf{Ag_{19}Cu_2}$ cluster and the molecular formulas of the corresponding species for fragment peaks.

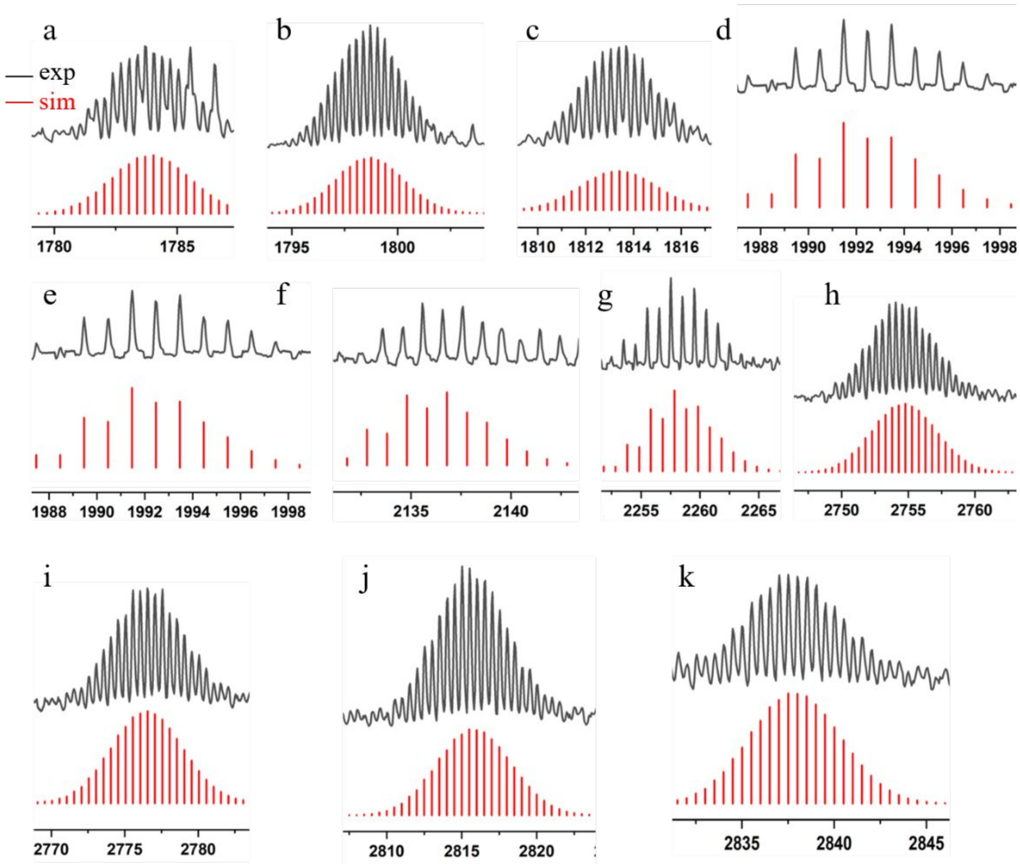

Figure S6. Mass spectra of $\mathbf{Ag_{19}Cu_2}$, the measured (black trace) and simulated (red trace) isotopic distribution patterns of the corresponding the molecular ion peaks.

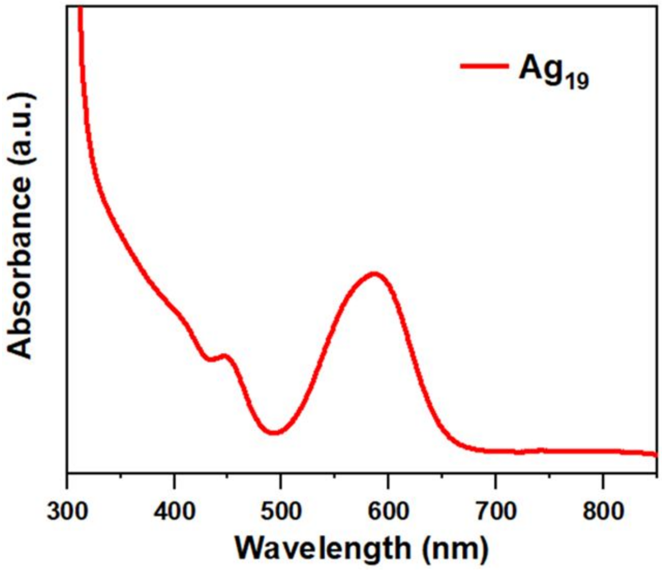

Figure S7. UV-vis absorption spectrum of the solution containing $AgSbF_6$, 4-^{t}BuPhC≡CH, Dpppe, and Et_3N under irradiation.

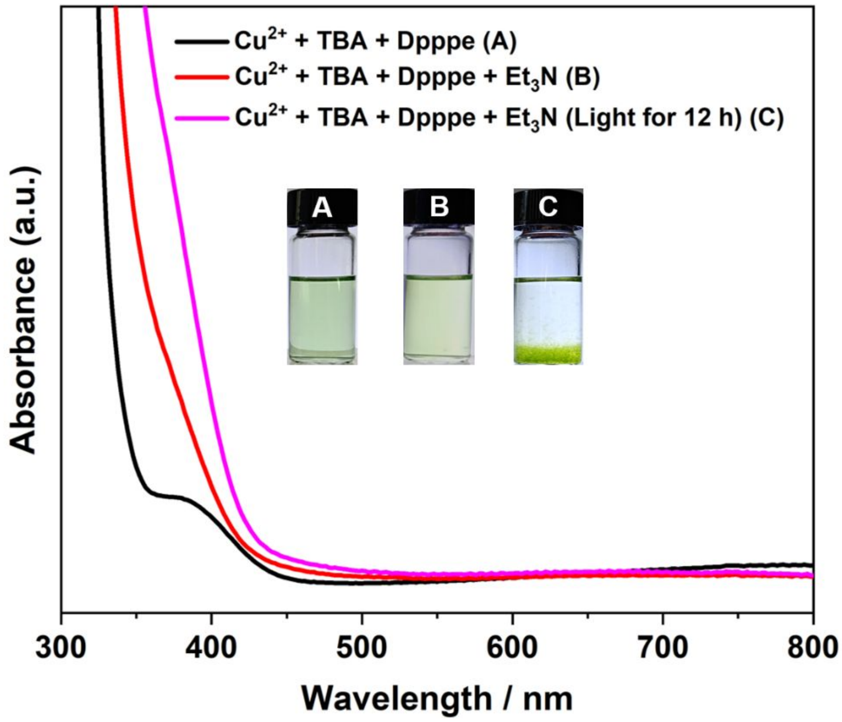

Figure S8. UV-vis spectra of the solution containing $Cu(CF_3COO)_2$ and other components after irradiation. The insets are corresponding photographs.

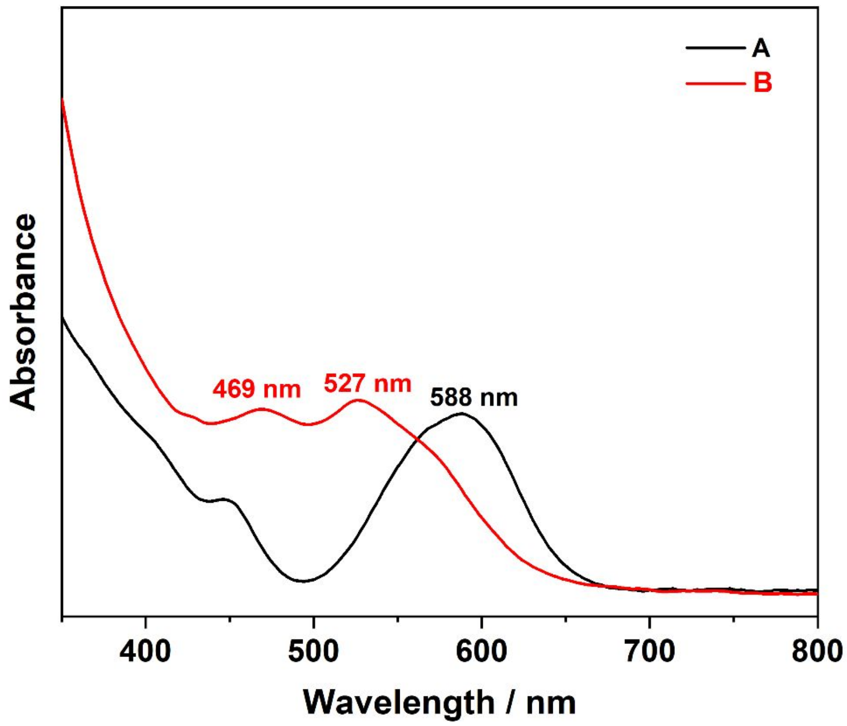

Figure S9. UV-vis absorption spectra of solutions. (A) $\mathbf{Ag_{19}}$ single crystals dissolved in CH_2Cl_2. (B) Introduce 0.1 equivalent $Cu(CF_3COO)_2$ into $\mathbf{Ag_{19}}$ solution and reaction for 5 minutes.

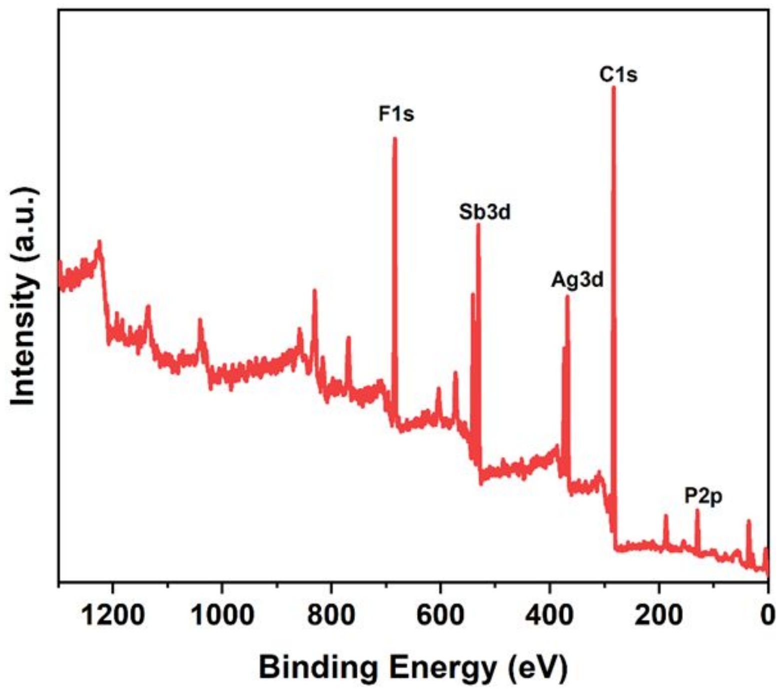

Figure S10. A Survey scan of XPS spectrum of $\mathbf{Ag_{19}Cu_2}$ nanocluster.

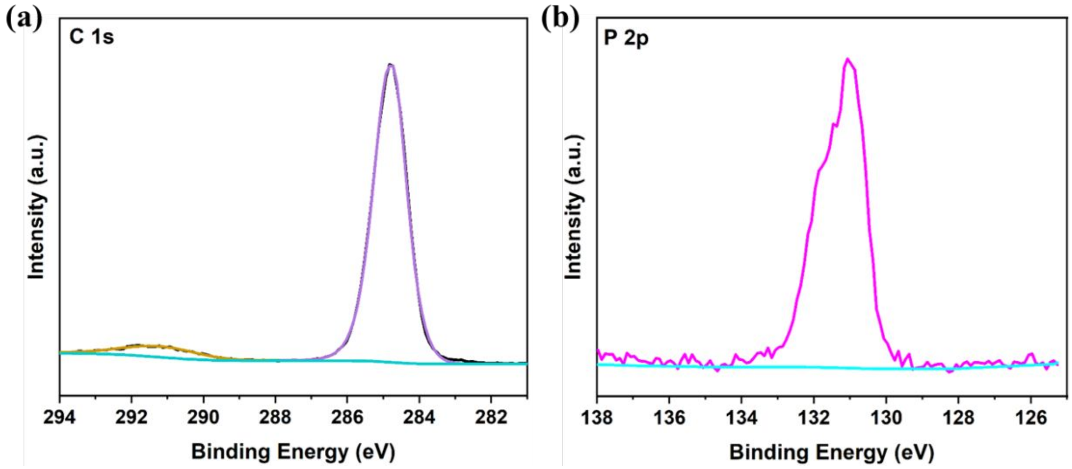

Figure S11. High-resolution XPS spectra of C 1s and P 2p for $\mathbf{Ag_{19}Cu_2}$ nanocluster.

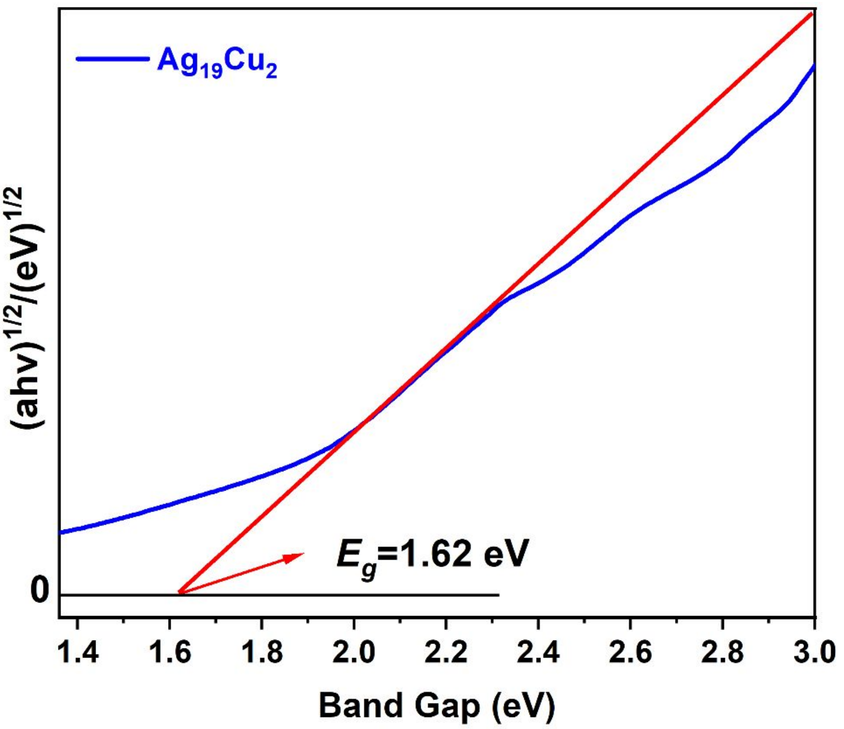

Figure S12. Band gap determined from the Tauc plot by the UV-vis absorption spectrum.

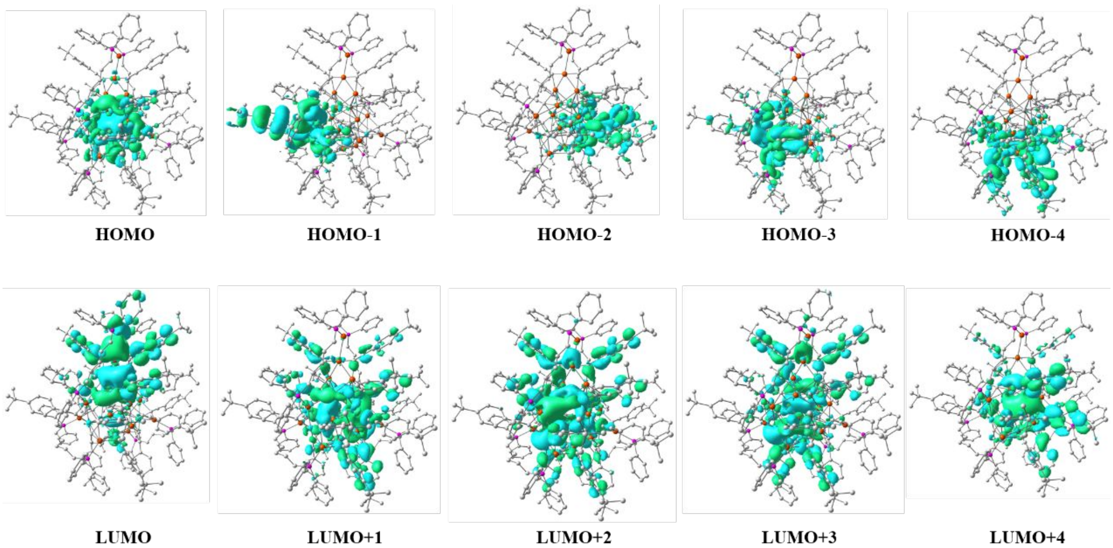

Figure S13. Calculated molecular energy levels (PBEPBE/DEF2SVP) of corresponding electronic charge densities for HOMOs and LUMOs.

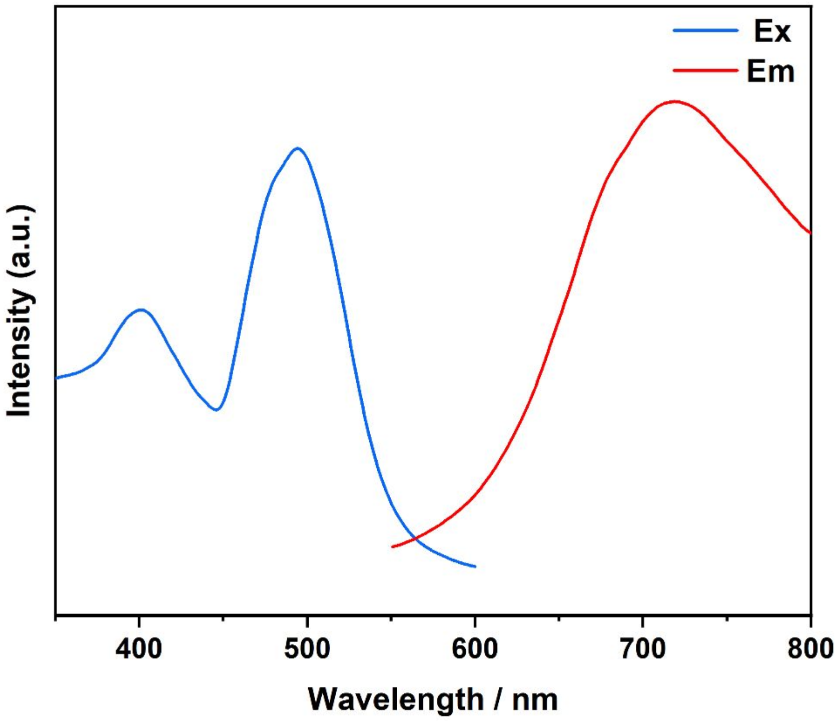

Figure S14. The photoluminescence characterization of $\mathbf{Ag_{19}Cu_2}$ powder.

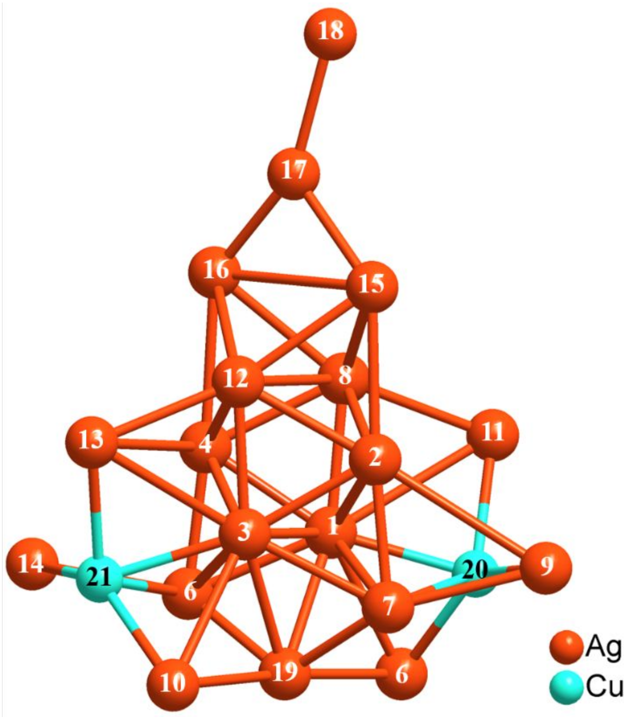

Figure S15. The atomic numbers of metal atoms in the $\mathbf{Ag_{19}Cu_2}$ structure.

**Atom**	Ag1	Ag2	Ag3	Ag4	Ag5	Ag6	Ag7	Ag8	Ag9	Ag10	Ag11
**Hirshfeld Charge**	0.067	0.167	0.071	0.171	0.186	0.190	0.193	0.158	0.189	0.184	0.185
**Atom**	Ag12	Ag13	Ag14	Ag15	Ag16	Ag17	Ag18	Ag19	Cu20	Cu21	
**Hirshfeld Charge**	0.158	0.183	0.185	0.213	0.212	0.184	0.128	0.187	0.228	0.227	

Figure S16. Summary of Hirshfeld Charge Analysis of $\mathbf{Ag_{19}Cu_2}$.

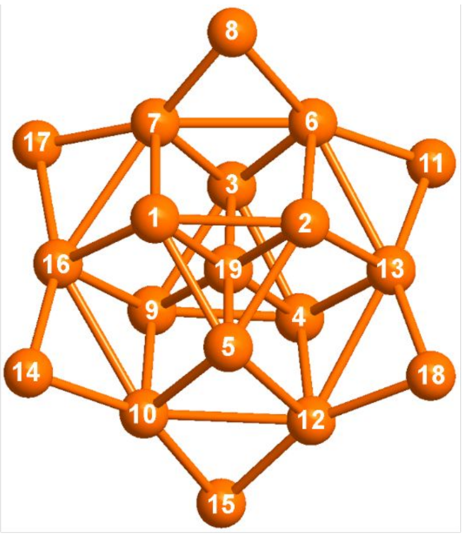

Figure S17. The atomic numbers of metal atoms in the $\mathbf{Ag_{19}}$ structure.

**Atom**	Ag1	Ag2	Ag3	Ag4	Ag5	Ag6	Ag7	Ag8	Ag9	Ag10
**Hirshfeld Charge**	0.174	0.173	0.171	0.172	0.172	0.130	0.135	0.174	0.174	0.136
**Atom**	Ag11	Ag12	Ag13	Ag14	Ag15	Ag16	Ag17	Ag18	Ag19	
**Hirshfeld Charge**	0.168	0.130	0.134	0.171	0.174	0.127	0.170	0.173	-0.03	

Figure S18. Summary of Hirshfeld Charge Analysis of $\mathbf{Ag_{19}}$.

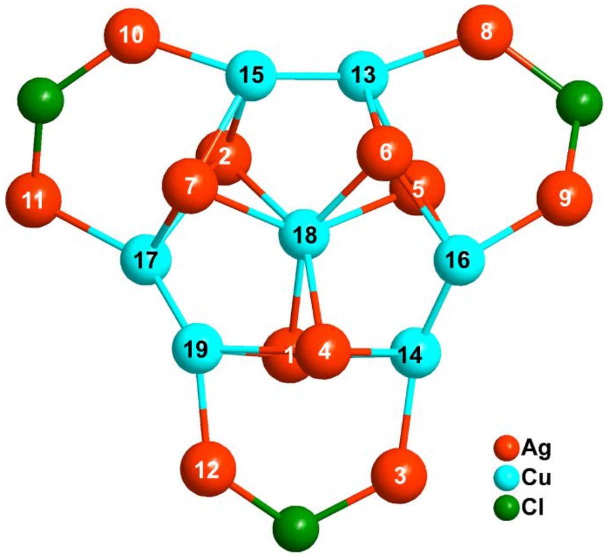

Figure S19. The atomic numbers of metal atoms in the $\mathbf{Ag_{12}Cu_7}$ structure.

**Atom**	Ag1	Ag2	Ag3	Ag4	Ag5	Ag6	Ag7	Ag8	Ag9	Ag10
**Hirshfeld Charge**	0.231	0.228	0.175	0.223	0.229	0.231	0.227	0.173	0.172	0.173
**Atom**	Ag11	Ag12	Cu13	Cu14	Cu15	Cu16	Cu17	Cu18	Cu19	
**Hirshfeld Charge**	0.165	0.169	0.209	0.215	0.210	0.211	0.201	0.207	0.194	

Figure S20. Summary of Hirshfeld Charge Analysis of $\mathbf{Ag_{12}Cu_7}$.

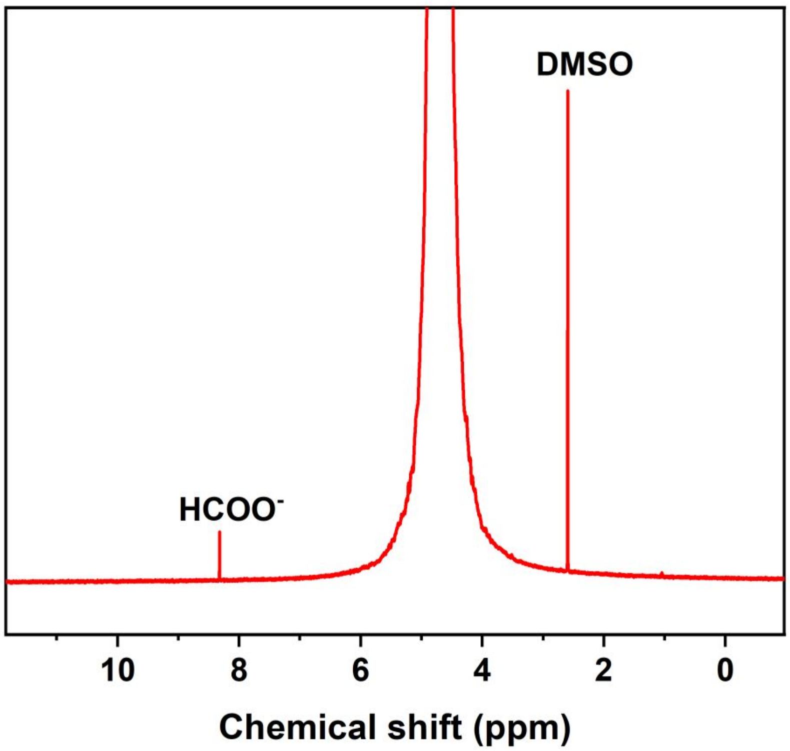

Figure S21. ¹H NMR spectrum of the electrolyte after 1 h CO_2 reduction electrolysis at -1.22 V vs. RHE in 0.5 M $KHCO_3$ solution.

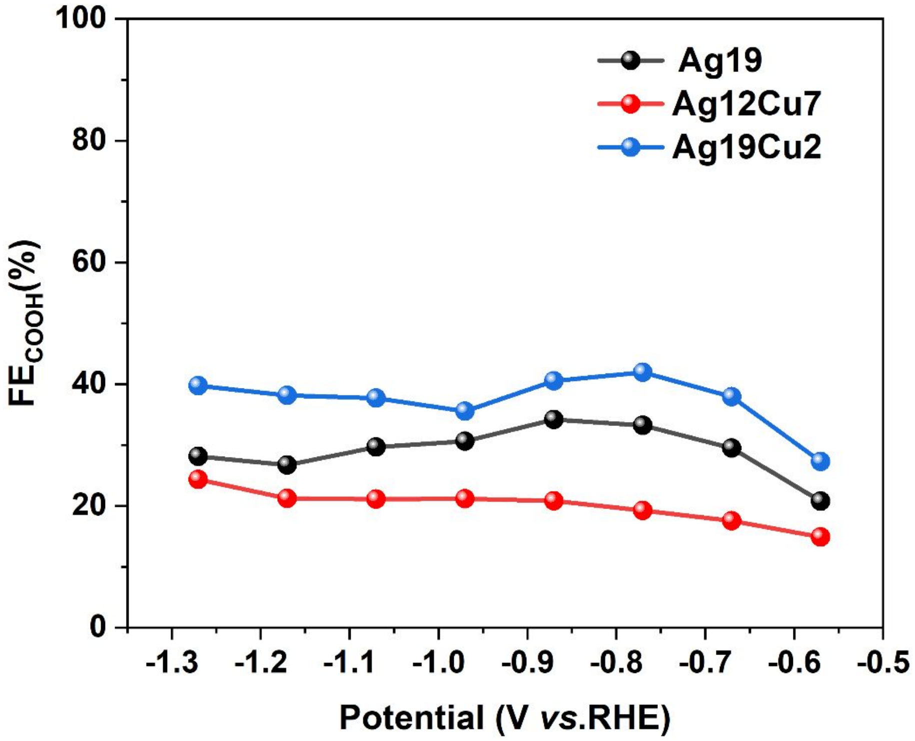

Figure S22. Faradaic efficiency of HCOOH examined at different applied potentials.

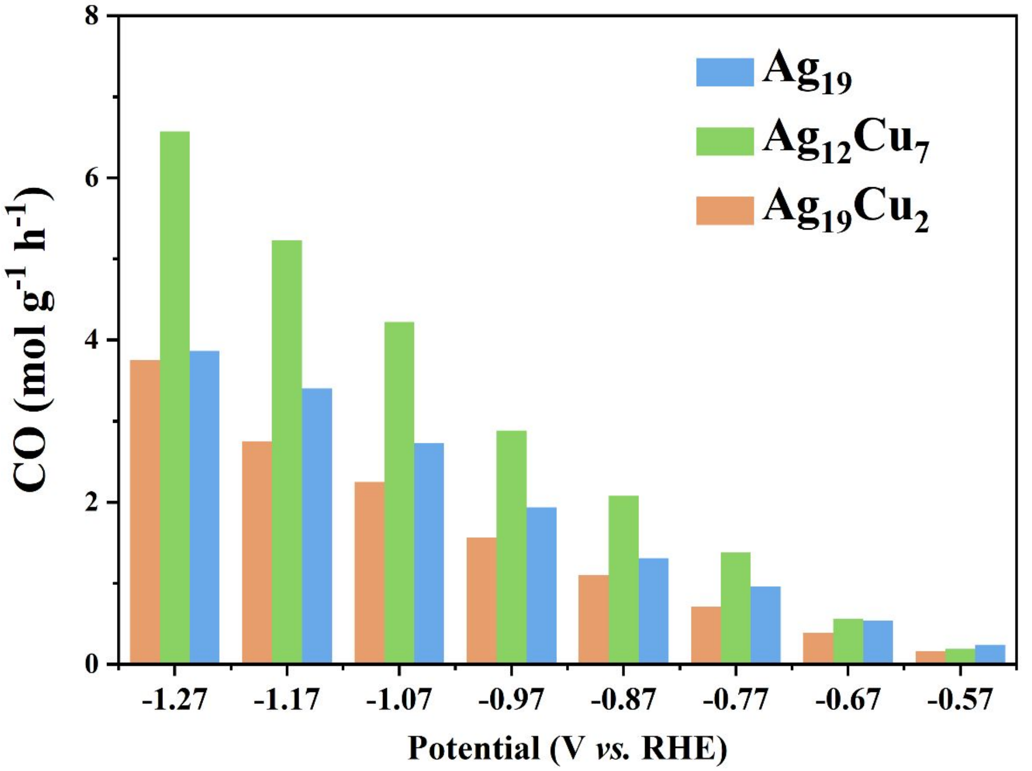

Figure S23. Production rate of CO.

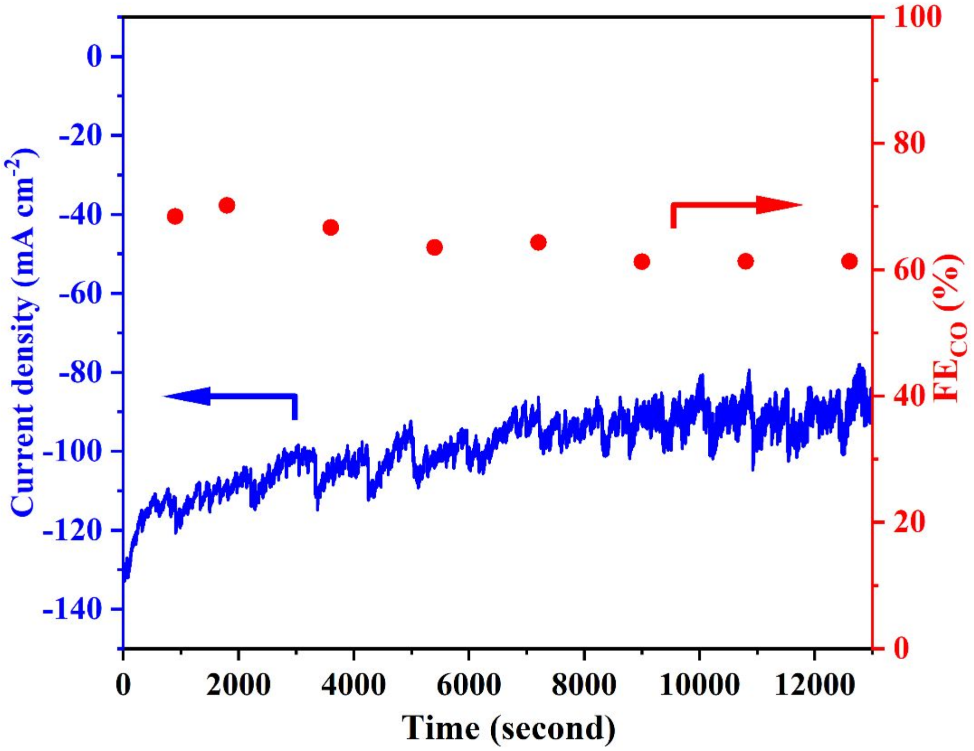

Figure S24. Long-term stability of $\mathbf{Ag_{12}Cu_7}$ at -0.97 V vs. RHE.

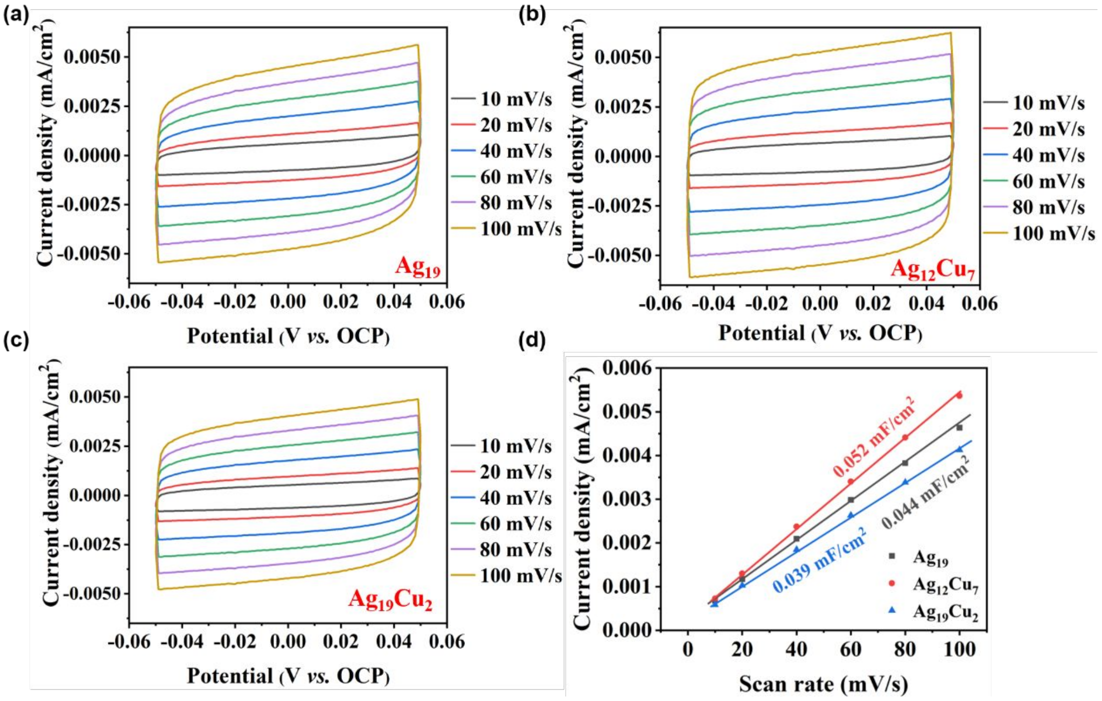

Figure S25. Electrochemical double-layer capacitance measurements. Cyclic voltammetry curves measured at different scan rates for (a) $\mathbf{Ag_{19}}$, (b) $\mathbf{Ag_{12}Cu_7}$ and (c) $\mathbf{Ag_{19}Cu_2}$. The scan rates are 10, 20, 40, 60, 80 and 100 mV s-1. (d) Slope analysis of electrochemically active surface area (ECSA)-electric double layer capacitance (Cdl) correlation derived from cyclic voltammetry measurements.

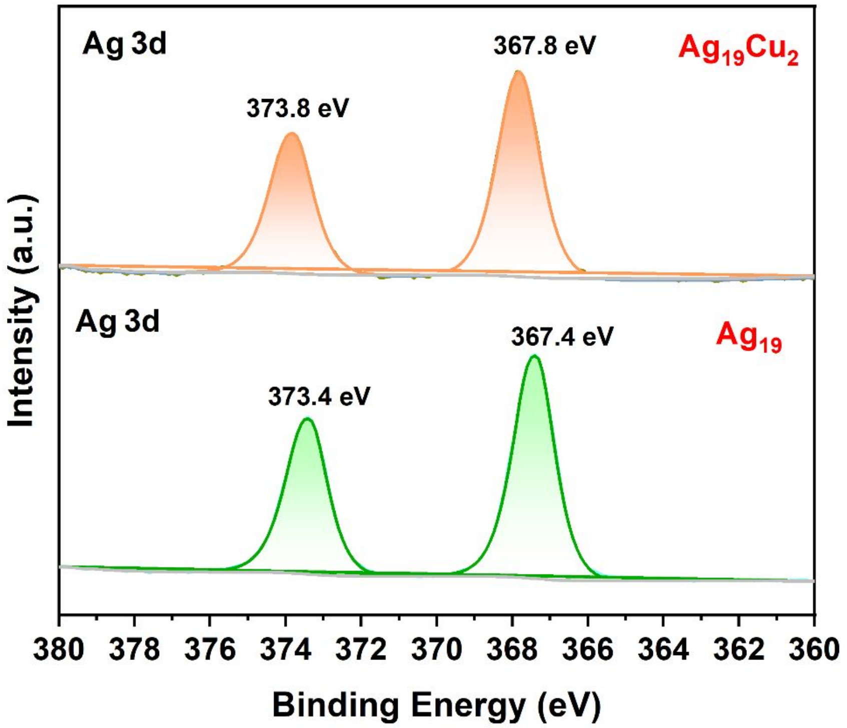

Figure S26. X-ray photoelectron spectroscopy (XPS) of Ag3d in $\mathbf{Ag_{19}}$ and $\mathbf{Ag_{19}Cu_2}$ nanoclusters.

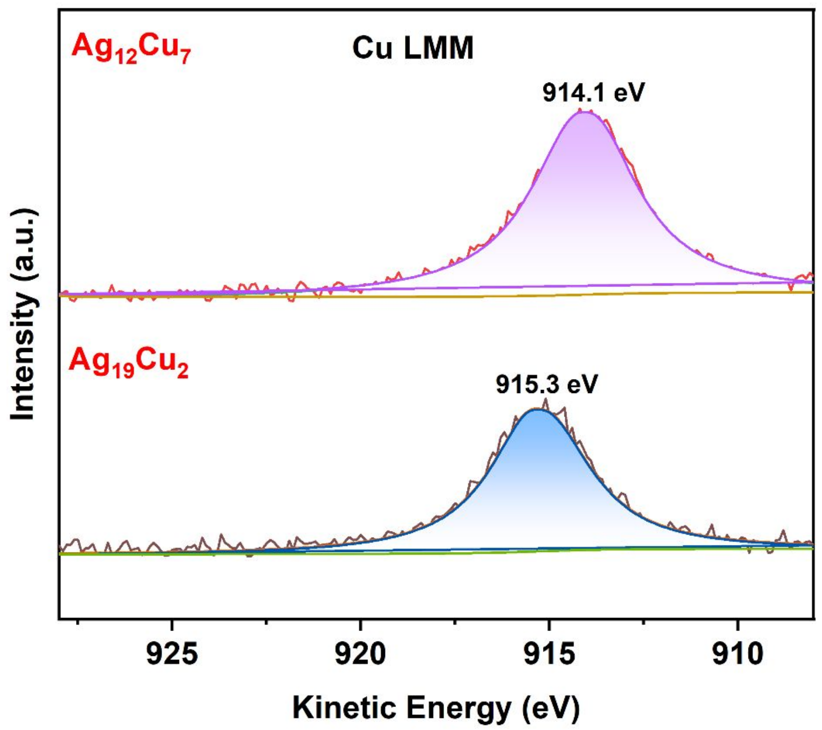

Figure S27. X-ray excited Cu LMM Auger electron spectroscopy of $\mathbf{Ag_{19}Cu_2}$ and $\mathbf{Ag_{12}Cu_7}$ nanoclusters.

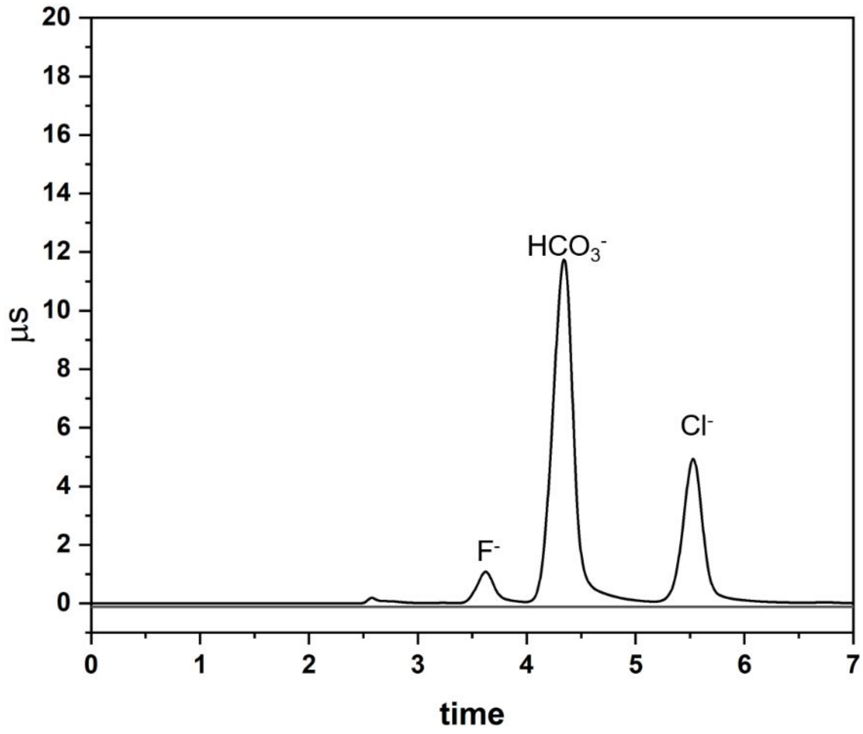

Figure S28. The extraction of the ion chromatogram of the electrolyte after the electrocatalytic CO_2 reduction reaction of $\mathbf{Ag_{12}Cu_7}$ nanocluster.

Table S1. Crystal data and structure refinement for **$Ag_{19}Cu_2$**.

Identification code	$[Ag_{19}Cu_2(4\text{-}^tBuPhC\equiv C)_{16}(Dpppe)_4](SbF_6)_3$
Empirical formula	$C_{308}H_{328}Ag_{19}Cu_2F_{18}P_8Sb_3$
Formula weight	7161.30
Temperature/K	150.1
Crystal system	triclinic
Space group	P-1
a/Å	21.9134(3)
b/Å	25.6539(3)
c/Å	32.0720(3)
α/°	96.7100(10)
β/°	97.8920(10)
γ/°	99.2380(10)
Volume/$Å^3$	17448.6(4)
Z	2
$\rho_{calc}g/cm^3$	1.363
μ/mm^{-1}	11.028
F(000)	7124.0
Crystal size/mm^3	0.2 × 0.25 × 0.3
Radiation	CuKα (λ = 1.54184)
2Θ range for data collection/°	7.048 to 119.998
Index ranges	$-24\le h\le 24, -28\le k\le 28, -36\le l\le 32$
Reflections collected	193115
Independent reflections	51751 [Rint = 0.0623, Rsigma = 0.0497]
Data/restraints/parameters	51751/2892/3397
Goodness-of-fit on F^2	1.051
Final R indexes [I>=2σ (I)]	R1 = 0.0625, wR2 = 0.1627
Final R indexes [all data]	R1 = 0.0740, wR2 = 0.1728
Largest diff. peak/hole / e $Å^{-3}$	2.73/-2.50

REFERENCES

1. CrysAlis^{Pro} Version 1.171.35.19. Agilent Technologies Inc. Santa Clara, CA, USA, **2011**.
2. Dolomanov, O. V., Bourhis, L. J., Gildea, R. J., Howard, J. A. K., Puschmann, H. OLEX2: a complete structure solution, refinement and analysis program. *J. Appl. Crystallogr*, **2009**, *42*, 339-341.
3. Fukazawa, Y., Rubtsov, A. E., Malkov, A. V. A Mild Method for Electrochemical Reduction of Heterocyclic N-Oxides. *Eur. J. Org. Chem.* **2020**, *2022*, 3317-3319.
4. Kresse, G., Furthmüller, J. Efficient iterative schemes for ab initio total-energy calculations using a plane-wave basis set. *Phys. Rev. B*, **1996**, *54*, 11169.
5. Hammer, B., Hansen, L. B., Nørskov, J. K. Improved adsorption energetics within density-functional theory using revised Perdew-Burke-Ernzerhof functionals. *Phys. Rev. B*, **1999**, *59*, 7413.
6. Blöchl, P. E. Projector augmented-wave method. *Phys. Rev. B*, **1994**, *50*, 17953-17979.
7. Chan, K., Tsai, C., Hansen, H.A. and Nørskov, J.K., Molybdenum Sulfides and Selenides as Possible Electrocatalysts for CO_2 Reduction.*ChemCatChem*, **2014**, *6*, 1899–1905.
